# Multisensory Integration in Stroke Patients: A Theoretical Approach to Reinterpret Upper-Limb Proprioceptive Deficits and Visual Compensation

**DOI:** 10.3389/fnins.2021.646698

**Published:** 2021-04-07

**Authors:** Jules Bernard-Espina, Mathieu Beraneck, Marc A. Maier, Michele Tagliabue

**Affiliations:** Université de Paris, INCC UMR 8002, CNRS, Paris, France

**Keywords:** stroke, eye-hand coordination, maximum likelihood principle, visual compensation, proprioception assessment, multisensory integration

## Abstract

For reaching and grasping, as well as for manipulating objects, optimal hand motor control arises from the integration of multiple sources of sensory information, such as proprioception and vision. For this reason, proprioceptive deficits often observed in stroke patients have a significant impact on the integrity of motor functions. The present targeted review attempts to reanalyze previous findings about proprioceptive upper-limb deficits in stroke patients, as well as their ability to compensate for these deficits using vision. Our theoretical approach is based on two concepts: first, the description of multi-sensory integration using statistical optimization models; second, on the insight that sensory information is not only encoded in the reference frame of origin (e.g., retinal and joint space for vision and proprioception, respectively), but also in higher-order sensory spaces. Combining these two concepts within a single framework appears to account for the heterogeneity of experimental findings reported in the literature. The present analysis suggests that functional upper limb post-stroke deficits could not only be due to an impairment of the proprioceptive system per se, but also due to deficiencies of cross-references processing; that is of the ability to encode proprioceptive information in a non-joint space. The distinction between purely proprioceptive or cross-reference-related deficits can account for two experimental observations: first, one and the same patient can perform differently depending on specific proprioceptive assessments; and a given behavioral assessment results in large variability across patients. The distinction between sensory and cross-reference deficits is also supported by a targeted literature review on the relation between cerebral structure and proprioceptive function. This theoretical framework has the potential to lead to a new stratification of patients with proprioceptive deficits, and may offer a novel approach to post-stroke rehabilitation.

## Introduction

Manual dexterity is highly specialized in humans (Lemon, 2008). Multimodal information from different sensory systems need to be combined to optimally control hand movements. Among them are vision, proprioception, touch, audition and graviception. Goal-oriented upper limb movements are planned and controlled using mainly vision and proprioception, which allow comparison of hand position with the location/orientation of the object to be reached and/or grasped.

In the context of brain lesions, such as in stroke, proprioceptive deficits are common (Connell et al., 2008; Kessner et al., 2016). These deficits significantly contribute to the patients’ motor disability and largely determine their degree of recovery (Turville et al., 2017; Zandvliet et al., 2020). Despite the clinical relevance, no consensus exists regarding the neurological assessment of proprioceptive deficits, nor on the rehabilitation strategies (Findlater and Dukelow, 2017). Clinical research studies that investigated and compared various techniques for the assessment of proprioception observed inconsistencies (Dukelow et al., 2012; Gurari et al., 2017; Ingemanson et al., 2019). Attempts to quantify the patients’ ability to use vision to compensate for proprioceptive deficits also provided contrasting results depending on the task and on the brain lesion (Darling et al., 2008; Scalha et al., 2011; Semrau et al., 2018; Herter et al., 2019).

In the present non-systematic review, we propose a new analysis and re-classification of assessment techniques commonly used in clinical practice and stroke research. This reinterpretation is based on the theoretical framework provided by the Maximum Likelihood Principle (MLP) and its application in the field of perception and sensorimotor control (Van Beers et al., 1996; Ernst and Banks, 2002; Körding et al., 2007). This theory describes how sensory inputs are optimally combined to generate a coherent movement representation and statistically maximize its precision. Experimental evidence, and its interpretation through this statistical model, suggests that the central nervous system (CNS) reconstructs multiple concurrent representations of the task (Tagliabue and McIntyre, 2008; McGuire and Sabes, 2009; Tagliabue and McIntyre, 2011, 2014). Each of these concurrent representations encodes the information in a specific reference frame, which can be directly associated to a sensory system (e.g., the retinal reference for vision and the joint reference for proprioception) or to a combination of sensory signals (i.e., body-centered, gravito-centered and allocentric references). As a consequence, the information acquired through a sensory channel can be encoded in a reference frame not directly associated to the originating sensory system. This information processing is commonly termed “cross-modal” when the transformations involves two reference frames associated to two different sensory modalities. In the following we will privilege the more generic “cross-reference” term, which accounts for both between-modalities transformations (e.g., proprioceptive to visual) and within-modality transformations (e.g., proprioceptive transformation between different reference frames as the hand or the trunk, or even with respect to external references).

Cross-reference processing appears to take place even when the constraints of the task leaves only one sensory input modality available (Pouget et al., 2002; Sarlegna and Sainburg, 2007; McGuire and Sabes, 2009; Jones and Henriques, 2010; Tagliabue and McIntyre, 2013; Arnoux et al., 2017). It is therefore critical to distinguish between the modality of the sensory inputs provided by the task, and the potential cross-reference sensory processing that ensues during task performance.

The present reinterpretation of the contrasting results reported in the stroke literature is founded on the hypothesis that altered cross-reference processing could form an essential part of what has (perhaps misleadingly) been termed proprioceptive post-stroke deficits.

In the next section, we will describe the standard methods used for the assessment of proprioceptive deficits and visual compensation mechanisms post-stroke. In the following section we will present the multisensory integration theory based on MLP and its application to the most representative clinical tests. Based on the MLP theoretical predictions, in section “Reinterpretation of Experimental Observations About Proprioceptive Deficits and Visual Compensation” we will propose a new stratification for stroke patients which is based on their sensory deficits. In section “Insights From Brain Lesions and Functional Anatomy Studies,” we will review lesion-behavior and brain imaging studies in the framework of this novel classification and attempt to relate brain structures to either purely proprioceptive functions or cross-reference processing. In the final section, we will summarize the contribution of this review to neuroscientific and clinical research and describe some specific applications for post-stroke sensory assessment and rehabilitation.

## Upper Limb Proprioceptive Deficits Post-Stroke

Stroke can affect not only motor abilities, but also sensory functions. In particular, proprioceptive deficits can be observed in a large percentage, up to 60%, of individuals following stroke (Connell et al., 2008; Kessner et al., 2016). These impairments are clearly correlated with functional deficits (Scalha et al., 2011; Meyer et al., 2014, 2016; Rand, 2018). In particular, reaching (Zackowski et al., 2004), dexterity (Carlsson et al., 2019), and inter-limb coordination (Torre et al., 2013) appear to be negatively affected by proprioceptive deficits. Moreover, sensory recovery is a predictive factor for functional recovery (Turville et al., 2017; Zandvliet et al., 2020).

Yet, no consensus seems to have emerged regarding proprioceptive assessment methods (Saeys et al., 2012; Simo et al., 2014; Pumpa et al., 2015; Santisteban et al., 2016). For the assessment of upper-limb function, no less than 48 different clinically validated (standardized) measures are used in clinical research (Santisteban et al., 2016). A high discrepancy between studies was found, as only 15 of the 48 outcome measures are used in more than 5% of the studies. In particular, only few studies specifically assess proprioceptive function: the NSA^1^, one of the most commonly used standardized scales, was applied in only 0.6% of studies reviewed (Santisteban et al., 2016). Moreover, current clinical practice does not systematically use standardized scales (Saeys et al., 2012; Simo et al., 2014; Pumpa et al., 2015; Santisteban et al., 2016; Matsuda et al., 2019). This lack of consensus is a major shortcoming for meta-analysis of recovery of upper limb function after stroke (Findlater and Dukelow, 2017). Similarly, research examining the ability of patients to compensate for a proprioceptive deficit using vision lack homogeneity. Although empirical evidence suggests that vision is helpful to compensate a proprioceptive deficit (Pumpa et al., 2015), the studies addressing this question are scarce and their methodologies are hardly comparable (Darling et al., 2008; Scalha et al., 2011; Torre et al., 2013; Semrau et al., 2018; Herter et al., 2019).

In the following subsections we will review the assessment techniques currently used in stroke for proprioceptive function, as well as for visual compensation. We will then discuss several studies showing that some of these proprioception and visual compensation tests might lead to different diagnostics. Finally, in the last subsection we will propose a new categorization of these tests with the aim of better understanding the origin of their different outcomes.

### Proprioceptive Tests in the Clinical Practice

All existing proprioceptive assessment methods are relevant from a functional point of view, but their differences pose a challenge for their comparability. The commonly used tests, both in clinical practice (Pumpa et al., 2015) and in clinical research are described below:

• **Thumb Localization Test (TLT)**: Assesses the ability of a subject to localize a body part (thumb). The physiotherapist positions the affected arm of the patient who then has to point, without vision, to the affected thumb with the other, less-affected hand (Dukelow et al., 2012; Meyer et al., 2016; Rand, 2018).
• **Up or Down Test (UDT)**: Assesses the ability of a subject to detect joint displacement direction. The physiotherapist moves a joint of the patient whose vision is occluded. The subject is then asked to report the up or down movement direction. This test is part of the FMA-UE^2^ and the RASP^3^ (Scalha et al., 2011; Saeys et al., 2012; Simo et al., 2014; Rand, 2018; Birchenall et al., 2019; Carlsson et al., 2019; Frenkel-Toledo et al., 2019; Kessner et al., 2019; Pennati et al., 2020; Zandvliet et al., 2020).
• **Mirror Position Test (MPT)**: Assesses the ability of a subject to perceive the angular configuration of a particular joint. The physiotherapist positions a joint of the patient’s affected arm in the absence of vision. The patient is then asked to mirror the position with the other, less-affected arm. This task can also be performed using a robotic device. This test is part of the NSA (Connell et al., 2008; Dukelow et al., 2010; Scalha et al., 2011; Iandolo et al., 2014; Ben-Shabat et al., 2015; Meyer et al., 2016; Gurari et al., 2017; Sallés et al., 2017; Findlater et al., 2018; Rinderknecht et al., 2018; Semrau et al., 2018; Herter et al., 2019; Zandvliet et al., 2020).
• **Bimanual Sagittal Matching Test (BSMT)**: Assesses the ability of the patients to reproduce with their free hand the trajectory/position of the affected hand which is passively driven by a robotic device along the sagittal plane (Torre et al., 2013).
• **Within-arm Position Test (WPT)**: Assesses the ability of a subject to perceive the angular configuration of one joint. A robot moves the arm of the patient to a position to be memorized and then back to the initial configuration. Subsequently, the subject is asked to move his/her arm to the remembered position (Dos Santos et al., 2015; Contu et al., 2017; Gurari et al., 2017).
• **Matching to a Visual Image (MV)**: Assesses the ability of a subject to localize in space his/her unseen arm or hand relative to a visual reference. A visual image, that could be a lever or a virtual hand with a given orientation, is shown to the subject. The subject is then asked, without visual feedback, to reproduce the same orientation with his/her hand. The vision of the hand can be occluded by a box covering the hand, or by wearing a virtual reality headset that leaves the subject’s hand non-rendered (Turville et al., 2017; Deblock-Bellamy et al., 2018).
• **Threshold Detection Test (TDT)**: Assesses the patient’s ability to detect hand displacements of various magnitudes. Using a robotic device, a joint (elbow, wrist, metacarpophalangeal) is first moved from a starting to a reference position. Then, a second movement from the starting position in the same direction, but not with the same amplitude, is operated by the robot. The subject is asked to assess whether the second movement was larger or smaller than first one. The threshold detection value is measured (Simo et al., 2014; De Santis et al., 2015; Rinderknecht et al., 2018; Ingemanson et al., 2019).
• **Finger Proprioception Test (FPT)**: Assesses the patient’s ability to detect whether the index finger is aligned (in flexion/extension) with the middle finger. The two fingers are passively moved by a robotic device in a crossing flexion/extension movement. For each finger-crossing movement, the patient is asked to report when the two fingers are directly aligned relative to each other (Ingemanson et al., 2019).
• **Motor Sequences Test (MS)**: Assesses the patient’s ability to localize a body part (fingers). The subject is asked to touch with the thumb pad (I) the other finger pads (II, III, IV, V) with eyes closed. Motor sequences with alternating movements between the thumb and the other fingers are used: for example, touching in the following order: I with II, I with III, I with IV, I with V (Scalha et al., 2011).
• **Reaching Test (RT)**: Assesses the patient’s ability to localize in space his/her unseen arm relative to a visual reference. A visual target (real or on a screen) is shown and the subject asked to reach to the memorized target, without visual feedback of the reaching hand (Scalha et al., 2011; Elangovan et al., 2019; Valdes et al., 2019).
• **Shape** or **Length Discrimination (SLD)**: Assesses the patient’s ability to discriminate object shapes and dimensions without vision. Different objects of familiar geometric shapes, everyday objects or segments of different lengths are presented to the patient whose vision is occluded. Either with passive movements (operated by a robotic device or a physiotherapist) or active movements, the patient interacts with the different objects. The subject is asked to report the perceived shape, object or length (Van de Winckel et al., 2012; De Diego et al., 2013; Metzger et al., 2014; Sallés et al., 2017; Turville et al., 2017; Matsuda et al., 2019; Carlsson et al., 2019).

Although each one of these tests involves proprioception, they are clearly different. For instance, some tests involve one articular chain only (UDT, TDT, WPT), whereas others involve two distinct articular chains (two arms for MPT and TLT or two fingers for FPT and MS). When two articular chains are involved, the patient is either asked to mirror the joint configuration (MPT, FPT), or to point to a body part (e.g., thumb of the affected arm: TLT and MS). It is noteworthy that some other tests do not rely on proprioceptive inputs only, but use visually remembered references (MV, RT, SLD).

### Different Proprioceptive Assessments, Different Outcomes

Experimental observations suggest that methodological differences between these tests can lead to different diagnostics (Hirayama et al., 1999; Dukelow et al., 2012; Gurari et al., 2017; Ingemanson et al., 2019). Similarly, the ability of patients to compensate the proprioceptive deficit with vision depends on the task considered (Darling et al., 2008; Scalha et al., 2011; Torre et al., 2013; Semrau et al., 2018; Herter et al., 2019). In the following we will detail and discuss some of the studies reporting differences between proprioceptive assessment techniques for stroke patients.

#### Within-Arm Position Test (WPT) vs. Mirror Position Test (MPT)

Gurari et al. (2017) characterized the ability of chronic stroke patients and healthy controls to match elbow flexion/extension positions using two approaches: the **MPT** performed with a physiotherapist vs. the **WPT** under robotic control. The large majority of stroke patients showed impairments in the mirror task, but no difference with the control group in the within-arm task. These different outcomes could be due to lateralized sensory deficits observed after stroke (Connell et al., 2008; Kessner et al., 2016) resulting in asymmetries that may affect the between-arms comparison in the mirror task, but not the unilateral within-arm task. A non-exclusive alternative explanation for the difference in performances may reside in stroke lesions that could have damaged brain networks specifically involved in the mirror but not in the within-arm task (Iandolo et al., 2018). This second hypothesis appears supported by the results of  Torre et al. (2013), where stroke patients performed the bimanual sagittal matching tests (BSMT). The accomplishment of BSMT does not require mirroring with respect to the body midline of the hand position, because both hands moved along the sagittal plane, close to each other. The precision of the patients in this study is similar to that observed in within-arm tasks (Dos Santos et al., 2015; Contu et al., 2017; Rinderknecht et al., 2018) and appears better than for the MPT (Herter et al., 2019; Ingemanson et al., 2019), suggesting that stroke lesions can affect the sensory processing necessary to mirror the hand position with respect to the body midline without affecting the between-arms communication per se.

#### Mirror Position Test (MPT) vs. Thumb Localization Test (TLT)

Outcomes of these two tests were only poorly correlated (Kenzie et al., 2017) and could not reliably identify a proprioceptive deficit within the same patients (Dukelow et al., 2012). Estimated prevalence of proprioceptive deficits using these two tests varied by a factor of two (Meyer et al., 2016). A clear difference between the two tasks, which might explain the different outcomes, is the use of a left/right symmetric (MPT) vs. an asymmetric joint configuration in the TLT. Studies on healthy subjects comparing analogous symmetric and asymmetric inter-manual proprioceptive tasks suggest that these tests differ by the way the joint information from the two arms is processed (Arnoux et al., 2017). Stroke lesions may differentially damage brain areas involved in the specific sensory processing characterizing symmetric and asymmetric tasks.

#### Thumb Localization Test (TLT) and Finger Proprioception Test (FPT) vs. Up or Down Test (UDT)

These comparisons showed poor correlations (Lanska and Kryscio, 2000; Ingemanson et al., 2019), and prevalence of proprioceptive deficits varied by a factor of three (Hirayama et al., 1999). The difference between the unimanual UDT and both the inter-manual TLT and FPT, which uses two fingers of the affected hand, suggests that the different outcomes do not originate from involving only the affected limb. A key difference between these tasks resides in using a single (UDT) vs. two articular chains (TLT and FPT). Research on healthy subjects, comparing analogous proprioceptive tasks, supports differential proprioceptive processing in these two situations (Tagliabue and McIntyre, 2013).

#### Within-Arm Position Test (WPT) vs. Reaching Test (RT)

Performance errors in the WPT were only poorly correlated with errors in the RT (Darling et al., 2008). This result is most likely due to the obvious difference in sensory modality: the target position is either memorized through proprioception (WPT) or through vision (RT). These tasks have been studied in healthy subjects and been shown to require different sensory processing (Tagliabue and McIntyre, 2011; Tagliabue et al., 2013).

### Different Visual Compensation Assessments, Different Outcomes

Several studies tested whether stroke patients could compensate for their proprioceptive deficits by using visual information. The results appear to be very different depending on the task under investigation.

Visual feedback of the hand appears to improve the patient’s performance in some tasks, such as the **Motor Sequences Test** (Scalha et al., 2011), and the **Reaching Test** (Darling et al., 2008). On the other hand, in a large-scale study where patients were assessed using a **Mirror Position Test**, up to 80% of patients with proprioceptive deficits were not able to improve their performance when visual feedback of both arms was available (Semrau et al., 2018; Herter et al., 2019). The important difference between Mirror Position Test and both Motor Sequences Test and Reaching Test, is the different way visual information can be used. In both tasks where vision significantly improves performance in patients, the hand (or finger) reaches the same spatial position of the target: the tasks can hence be accomplished by simply matching the visually acquired target position and the visual feedback of the hand (or finger). In the Mirror Position Test in contrast, the patient does not have to reach the spatial location of the target, but its mirror position: the patient must thus “flip,” relative to the body midline, the image of the arms to evaluate the task accomplishment. It follows that the ability to use visual information to compensate for proprioceptive deficits in reaching, but not in mirror tasks, could be due to specific difficulties in performing “mirroring” of visual information. Consistent with this interpretation, patients were shown to be able to significantly improve their performance with vision in the Bimanual Sagittal Matching Test which does not require the “mirroring” of visual information, because their hands moved parallel to the sagittal plane and close to each other (Torre et al., 2013).

### Categorization of Proprioceptive Assessments

**FIGURE 1:**
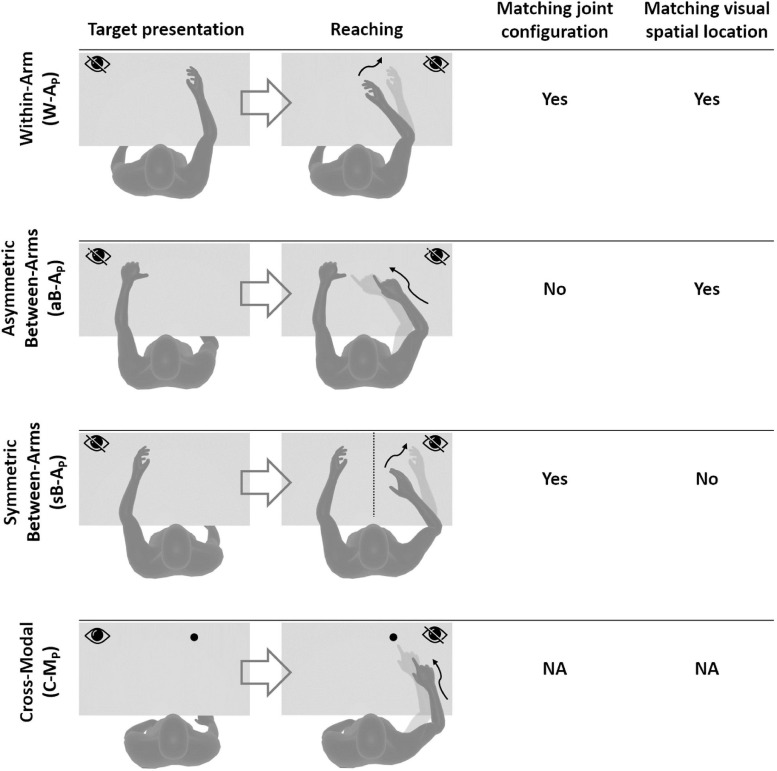
Four categories of proprioceptive assessments. In all represented examples, subjects are asked to, first, perceive a target position and then to reach for it. The last two columns show that the tasks categorization is based on the possibility, or not, to compare the target and effector position in joint and/or retinal space. In the within-arm category (W-A) the patient first perceives and then moves back to the target with the same arm. In the asymmetric between-arms category (aB-A) the location of the target perceived with one hand is subsequently reached with the other hand. In the symmetric between-arms category (sB-A) the patient perceives the target with one hand and mirrors its position with the other hand. In the cross-modal (C-M) category, where the hand and the target do not share the same sensory modality, the patient reaches for a visually memorized target with the unseen hand.

Based on the above observations, we propose here a new categorization of these various proprioceptive tests. We group them into four distinct categories (**within-arm** tasks, **asymmetric between-arms** tasks, **symmetric between-arms**^4^ tasks, and **cross-modal** tasks). This categorization is based on the possibility to achieve the tasks by reproducing the joint configuration memorized during the target acquisition and/or by matching the target position in retinal coordinates (Figure 1).

**Within-Arm tasks** require one and the same articular chain to perceive and to reproduce the target position. Thus, proprioceptive information to be remembered (target) and the feedback about the moving hand (effector) originate from the same joints (Figure 1, W-A). These tasks can be performed by directly matching the proprioceptive signals corresponding to the target and effector positions (Within-arm Position Test) or by directly comparing two movement signals originating from the same joints (Up or Down Test, Threshold Detection Test). These tasks can also be performed by matching the target and effector position encoded in the retinal reference. Bi-manual matching tests performed along the mid-sagittal plane (BSMT) are also associated to this category, because, as described in sections “Different Proprioceptive Assessments, Different Outcomes” and “Different Visual Compensation Assessments, Different Outcomes,” although involving two arms, the experimental results suggest that they are performed by a direct encoding of the information in joint and retinal coordinates, similarly to the within-arm tasks.

**Asymmetric Between-Arms tasks** involve two articular chains. Typically, the less-affected arm (effector) has to reach the target location perceived with the affected arm (Thumb Location Test, see Figure 1, aB-A). These tasks cannot be performed by matching the joint configuration of the affected arm (target) with that of the effector, since they differ at the end of the movement. They can be accomplished, however, by matching the target and effector location encoded in the retinal reference frame. The Motor Sequences test (involving only one arm), as well as the Thumb Location Test, can also be classified in this category since they involve different articular chains (fingers) to perceive the target position and to match it.

**Symmetric Between-Arms tasks** also involve two articular chains. “Symmetric” refers to the fact that the effector has to “mirror” the target configuration. The articular chains can be the arms (Mirror Position Test, see Figure 1, sB-A) or the index and middle fingers (Finger Proprioception Test). At task achievement, the joint configuration of the two articular chains is identical, allowing for direct matching of proprioceptive signals corresponding to the target and effector positions. In contrast, the task cannot be performed in the retinal space, since the target and the effector do not share the same spatial location.

**Cross-Modal Tasks** differ from the other three categories in that the target information is given visually (or remembered visually) whereas only proprioceptive information is provided for the effector (the moving hand, Figure 1, C-M). Thus, these tasks always require cross-reference sensory processing. For this reason, their categorization based on the direct encoding in the joint and/or retinal space is not fully applicable. Both Reaching Test and Matching to a Visual image share this characteristic. Similar sensory processing could also be involved in the tasks used in Perfetti’s neurocognitive approach, such as the Shape or Length Discrimination test.

Overall, this new categorization (summarized in Table 1) allows to discriminate the above-mentioned tests in terms of sensory requirements. In the following section, we will present the multisensory integration theory based on MLP and its application to the most representative clinical tests among those reported here.

**TABLE 1:** Categorization of proprioceptive assessments.

**Category**	**Test**
Within-arm (W-A)	Within-arm Position Test (WPT)
	Up or Down Test (UDT)
	Threshold Detection Test (TDT)
	Bimanual Sagittal Matching Test (BSMT)
Asymmetric between-arms (aB-A)	Thumb Localization Test (TLT)
	Motor Sequences Test (MS)
Symmetric between-arms (sB-A)	Mirror Position Test (MPT)
	Finger Proprioception Test (FPT)
Cross-modal (C-M)	Reaching Test (RT)
	Matching to a Visual image (MV)
	Shape/Length Discrimination (SLD)

## Optimal Multisensory Integration Theory and Stroke

In this section we will present the MLP and its application to generic target-oriented movements (first subsection). Then we will use this theoretical framework to describe the information processing underlying the proprioceptive assessments according to their categorization (second subsection).

### Statistical Optimality in Multisensory Integration for Goal-Oriented Hand Movements

When reaching to grasp an object, visual and proprioceptive sensory information about the target and the hand (effector) is used to control movement execution. In a first step, each sensory modality is encoded in the reference frame of the respective receptors: retinal and joint reference for vision and proprioception, respectively. Several studies have shown that redundant sensory signals are then optimally combined and weighted according to MLP in order to statistically minimize the variability of the estimated movement parameters (Ernst and Banks, 2002).

**FIGURE 2:**
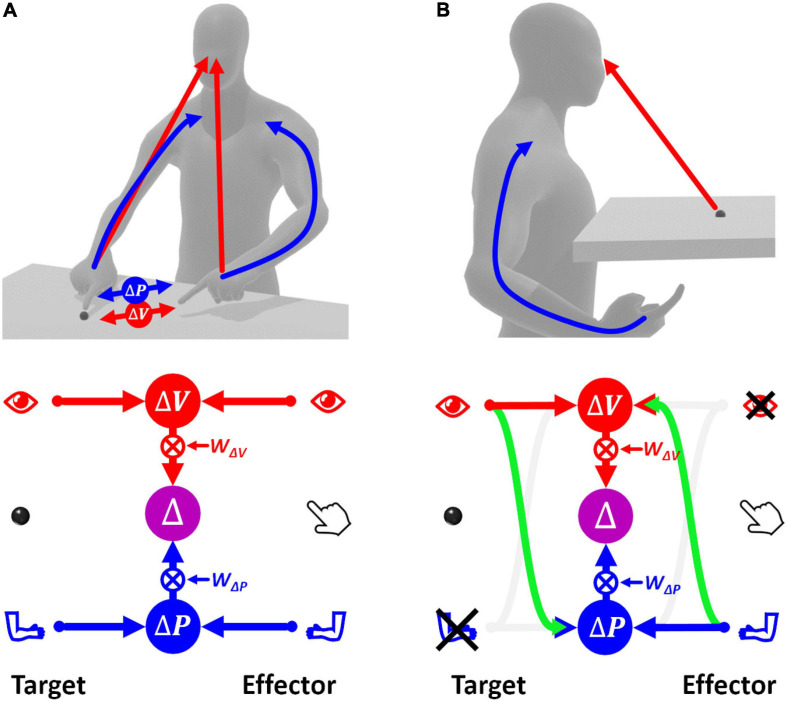
Concurrent Model of sensorimotor integration. In the bottom diagrams the left part represents the target information; the right part represents effector information. Target-effector comparisons are concurrently performed in visual (*V*) and proprioceptive (*P*) space. These two comparisons are then combined, using the relative weights *w_V_* and *w_P_*, leading to the optimal estimation of the motor vector. **(A)** Sensory information flow when the hand and target position are perceived through vision and proprioception concurrently. **(B)** Model prediction when the target position is perceived visually and the effector position is sensed through proprioception only. None of the two concurrent comparisons can be computed directly. In this condition, the model postulates occurrence of cross-reference transformations (green curved arrows) between sensory modalities. Blue: proprioceptive information, red: visual information, violet: multimodal visuo-proprioceptive processing.

Figure 2A shows how sensory signals are conceptually processed for goal-oriented upper limb movements. To match the target position with the effector, that is to reach the target with the hand, the latter must be displaced by a distance and in a direction that are represented by the movement vector Δ. To compute Δ, the target and effector positions are compared concurrently in the visual, *v*, and proprioceptive, *p*, space (Tagliabue and McIntyre, 2011). This is represented by the following equations of the visual and proprioceptive target-effector comparisons *v* and *p*:

(1) $$\begin{array}{c} \Delta \,V=x_{T,v}-x_{E,v}\\ \Delta \,P=x_{T,p}-x_{E,p} \end{array}$$

where *T* and *E* subscripts indicate an information about the target and the effector, respectively. For each sensory modality, the comparison is characterized by a variance corresponding to the sum of the variances of the target and effector information (Eq. 2).

(2) $$\begin{array}{c} \sigma_{\Delta \,V}^{2}=\sigma_{T,v}^{2}+\sigma_{E,v}^{2}\\ \sigma_{\Delta \,P}^{2}=\sigma_{T,p}^{2}+\sigma_{E,p}^{2} \end{array}\;$$

The MLP predicts that in order to maximize the precision of the estimated movement vector Δ, the concurrent visual and proprioceptive comparisons must be combined (summed), as in Eq. 3.

(3) $$\begin{array}{c} \Delta =w_{\Delta \,V}\cdot \Delta \,V+w_{\Delta \,P}\cdot \Delta \,P\\ w_{\Delta \,V}=\frac{\sigma_{\Delta \,P}^{2}}{\sigma_{\Delta \,V}^{2}+\sigma_{\Delta \,P}^{2}}\\ w_{\Delta \,P}=\frac{\sigma_{\Delta \,v}^{2}}{\sigma_{\Delta \,V}^{2}+\sigma_{\Delta \,P}^{2}} \end{array}$$

Thus, the movement vector is the weighted sum of the concurrent target-effector comparisons, and each comparison is associated to a weight, *w*_Δ*V*_ and *w*_Δ*P*_, whose value depends on the relative variability of the two comparisons.

If this MLP formulation, called “Concurrent Model,” is straightforward when both target and effector positions can be perceived through vision and proprioception (Figure 2A), the information processing seems more complex when some information is not available, e.g., when the target position can be perceived only visually while the effector position only through proprioception (Figure 2B). In this case, none of the two concurrent comparisons can be computed directly, because the target and the effector cannot be perceived through the same sensory modality. However, these comparisons can be performed through two mutually not exclusive possibilities: first, the visually perceived position of the target may be encoded in a proprioceptive space; second, the effector position, provided through proprioception, may be encoded in visual space.

In this condition the variability associated with the two concurrent comparisons is given in Eq. 4 where $\sigma_{p\to v}^{2}$ and $\sigma_{v\to p}^{2}$ represent the variance associated with the cross-reference transformations from proprioception to vision, and vice-versa. The indentation is used to facilitate the distinction between the variance associated with the target and effector encoding (the same type of indentation will be used throughout).

(4) $$\begin{array}{cccc} | & \mathrm{Target} & | & \mathrm{Effector}\\ \sigma_{\text{Δ}\,V}^{2}=\sigma_{T,v}^{2} & & +\sigma_{E,p}^{2}+\sigma_{p\to v}^{2} & \\ \sigma_{\text{Δ}\,P}^{2}=\sigma_{T,v}^{2}+\sigma_{v\to p}^{2} & & +\sigma_{E,p}^{2} & \end{array}$$

In contrast to the task represented in Figure 2A and Eq. 3, in this condition the two concurrent comparisons are not fully independent, because they are partially computed from the same information. In this case, Eq. 3 must be modified to take into account the covariance between proprioceptive and visual target-effector comparisons, *cov*(△*P*,△*V*) (see Supplementary Section 1 for details):

(5) $$\begin{array}{c} w_{\Delta \,V}=\frac{\sigma_{\Delta \,P}^{2}-c\,o\,v\,\left(△\,P,△\,V\right)}{\sigma_{\Delta \,V}^{2}+\sigma_{\Delta \,P}^{2}-2\cdot c\,o\,v\,\left(△\,P,△\,V\right)}\\ w_{\Delta \,P}=\frac{\sigma_{\Delta \,V}^{2}-c\,o\,v\,\left(△\,P,△\,V\right)}{\sigma_{\Delta \,V}^{2}+\sigma_{\Delta \,P}^{2}-2\cdot c\,o\,v\,\left(△\,P,△\,V\right)} \end{array}$$

For the example of Figure 2B$c\,o\,v\,\left(△\,P,△\,V\right)=\sigma_{T,v}^{2}$ + $\sigma_{E,p}^{2}$, that is the common variance component between $\sigma_{\Delta \,P}^{2}$ and $\sigma_{\Delta \,V}^{2}$. Therefore, Eq. 5 become:

(6) $$\begin{array}{c} w_{\Delta \,V}=\frac{\sigma_{v\to p}^{2}}{\sigma_{v\to p}^{2}+\sigma_{p\to v}^{2}}\\ w_{\Delta \,P}=\frac{\sigma_{p\to v}^{2}}{\sigma_{v\to p}^{2}+\sigma_{p\to v}^{2}} \end{array}$$

It follows that the relative weights between the two concurrent object-effector comparisons depend on the noisiness of the two cross-modal transformations, which is consistent with experimental observations (Burns and Blohm, 2010; Tagliabue et al., 2013).

### Application of the Optimal Sensory Integration Theory to Proprioception Assessment Tests

In the following we will show whether the MLP predicts clear differences between the sensory processing necessary to accomplish the tasks depending on their categorization described in the previous section.

**FIGURE 3:**
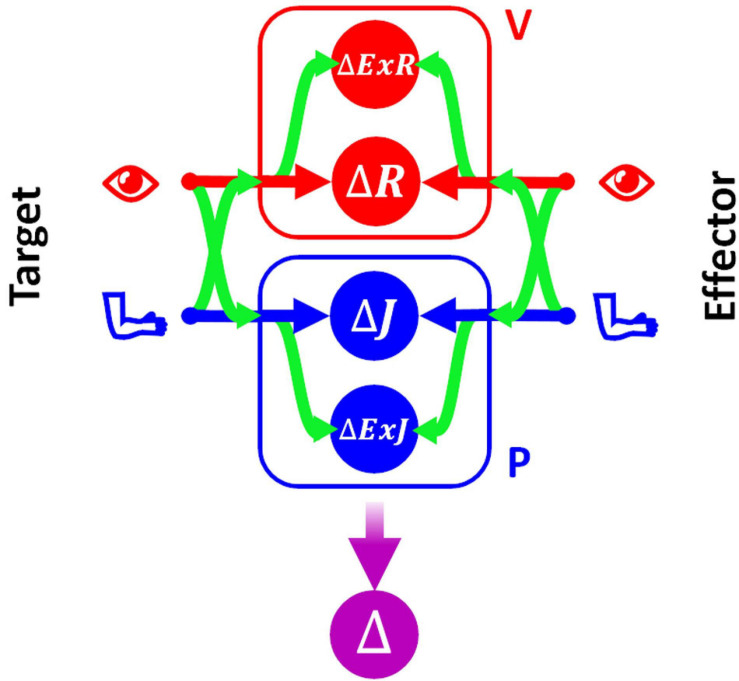
Concurrent Model for proprioceptive assessment tasks. Sensory inputs from the target and the effector are concurrently compared in four reference-frames: retino-centered (Δ*R*), joint-centered (Δ*J*), extra-retinal (Δ*ExR*), and extra-joint (Δ*ExJ*). The visual (red) and proprioceptive (blue) signals are primarily encoded in retino-centered and joint-centered reference frames, respectively, but they can also be encoded in additional “secondary” reference frames not directly associated with the respective receptors. To encode a sensory signal in a secondary reference frame, cross-reference transformations (represented by the curved green arrows) are necessary.

In order to be able to represent consistently the type of sensory processing underlying the execution of tasks within these four categories, we will use a slightly modified formulation of the Concurrent Model with respect to the one presented in section above. This formulation, represented in Figure 3, explicitly distinguishes between the reference frames in which the sensory signals are natively encoded (the joint, *J*, and the retinal, *R*, reference frames for proprioception and vision, respectively) and the reference frames which correspond to a combination of the original sensory signal about target and response position, with additional sensory information. For instance, the hand position perceived through joint receptors can be encoded with respect to different body parts or even with respect to external references, such as gravity or visual landmarks (Tagliabue and McIntyre, 2014). To refer to this type of indirect sensory encodings we use the generic term “extra-joint,” *ExJ*, for proprioception and “extra-retinal,” *ExR*, for vision.

Although both visual and proprioceptive information can potentially be encoded in multiple “extra-” reference frames, we have reduced the model formulation to its simplest version allowing an accurate description of the sensory processing underlying the analyzed tasks. As a consequence, the present formulation of MLP includes four concurrent target-effector comparisons: △*J*, △*R*, △*ExJ*, △*ExR*. In this formulation of the concurrent model the estimation of the motor vector △ corresponds to the following weighted sum:

(7) $$\Delta =w_{\Delta \,J}\,\Delta \,J+w_{\Delta \,E\,x\,J}\,\Delta \,E\,x\,J+w_{\Delta \,R}\,\Delta \,R+w_{\Delta \,E\,x\,R}\,\Delta \,E\,x\,R$$

To represent all possible cross-reference transformations between these four reference frames, this model includes not only the possibility to perform cross-reference transformations between proprioceptive, joint-centered, and visual, retino-centered reference frames (*J*↔*R*), but also the possibility to encode joint and retinal signals in the extra-joint and extra-retinal reference frames, respectively (*J*→*ExJ* and *R*→*ExR*).

In the following this statistical model will be used to evaluate, for each of the categories of proprioceptive assessments, the relative weights that must be associated with the four concurrent target-effector comparisons to optimize the precision of the movement vector estimation, Δ. The precise values of the sensory weight and details of the methods used are reported in Supplementary Sections 2, 3. In the following paragraphs these results will be only graphically described in the figures representing the information flow theoretically associated with each category of tasks. The analytical equation of the variability of the optimal motor vector estimation predicted by MLP will be reported for each test and will then be quantitatively compared to the results of experimental studies.

#### Within-Arm Proprioceptive Tasks (W-A_*P*_)

In this test the memorized target and the effector positions are perceived through the same set of joint sensors. Thus, their position can be compared “directly” in the joint space *J*. All three other concurrent comparisons would require some cross-reference transformation. The variance associated with each of the four concurrent target-response comparisons for the W-A_*P*_ tasks is reported in Eq. 8, where $\sigma_{J\to R}^{2}$ is the variance associated with the cross-reference transformation from the joint-centered to the retino-centered reference frame. $\sigma_{J\to E\,x\,J}^{2},\sigma_{R\to E\,x\,R}^{2}$ are the variances corresponding to the intra-modal transformations from joint to extra-joint and from retinal to extra-retinal references, respectively.

(8) $$\begin{array}{ccc} \sigma_{△\,J}^{2} & =\sigma_{J}^{2} & +\sigma_{J}^{2}\\ \sigma_{△\,E\,x\,J}^{2} & =\sigma_{J}^{2}+\sigma_{J\to E\,x\,J}^{2} & +`\,\sigma_{J}^{2}+\sigma_{J\to E\,x\,J}^{2}\\ \sigma_{△\,R}^{2} & =\sigma_{J}^{2}+\sigma_{J\to R}^{2} & +\sigma_{J}^{2}+\sigma_{J\to R}^{2}\\ \sigma_{△\,E\,x\,R}^{2} & =\sigma_{J}^{2}+\sigma_{J\to R}^{2}+\sigma_{R\to E\,x\,R}^{2} & +\sigma_{J}^{2}+\sigma_{J\to R}^{2}+\sigma_{R\to E\,x\,R}^{2} \end{array}$$

**FIGURE 4:**
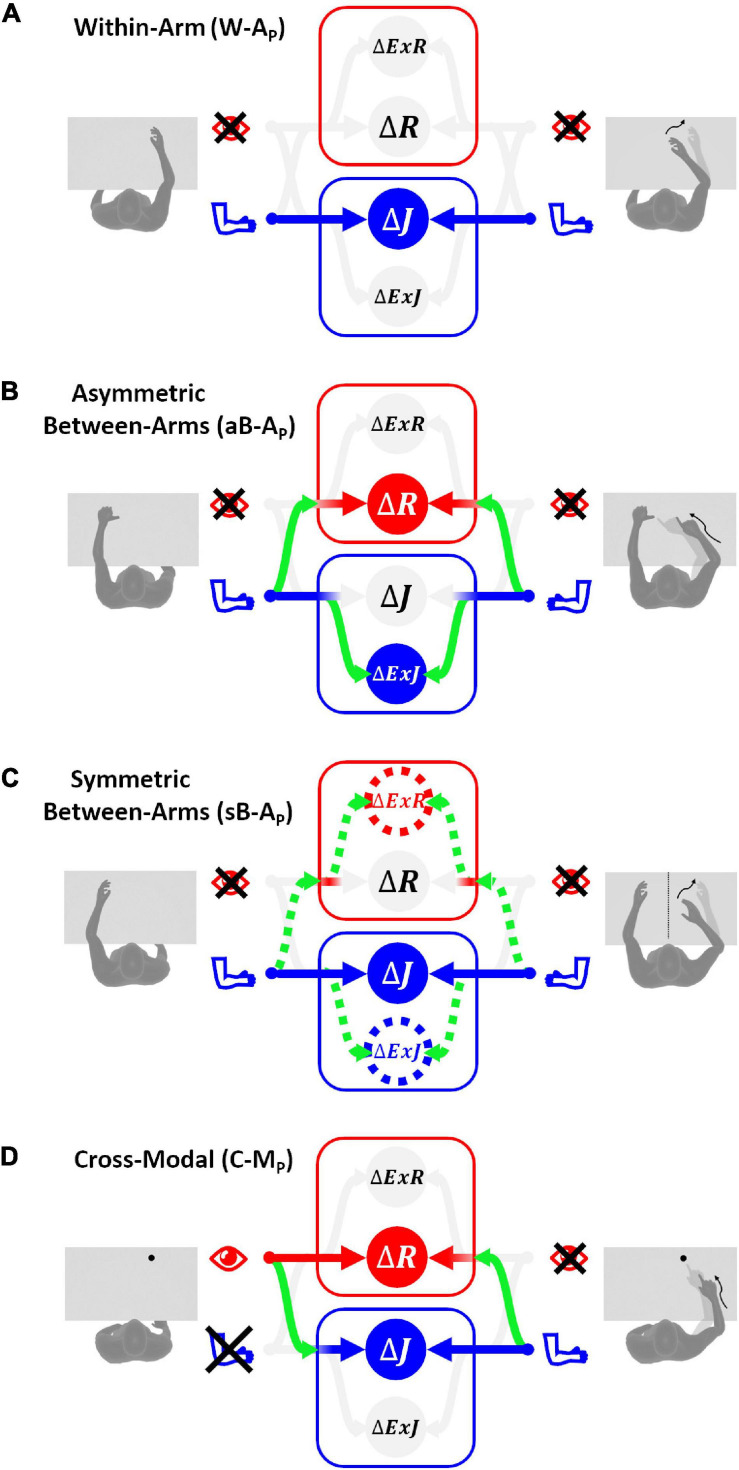
Sensory information flow predicted for proprioceptive tests. The model results are reported separately for the four categories of tests without vision of the arms: **(A)** within-arm task (W-A_*P*_), proprioceptive joint signals from the right arm during the target memorization (left column) can be directly compared with proprioceptive joint signals from the same arm during the response phase (right column). **(B)** Asymmetric between-arms task (aB-A_*P*_), the task cannot be achieved by simply matching the homologous proprioceptive joint signals from the left and right arm: the use of alternative reference frames and cross-reference transformations (green curved arrows) is necessary. **(C)** Symmetric between-arms task (sB-A_*P*_), proprioceptive joint signals from the left arm during the target memorization can theoretically be compared directly with the homologous proprioceptive joint signals from the right arm. However, for patients with inter-hemispheric transformation impairment, indirect comparisons (doted lines) are necessary. **(D)** Cross-modal task (C-M_*P*_), the target and the effector do not share the same sensory modality. The model prediction in this situation consists in encoding the task in both joint and retinal space by performing the depicted cross-reference transformation. The target-effector comparisons performed in a sensory space associated with a weight close to zero are in pale gray, while those associated with weights significantly larger than zero are in bright colors.

The optimal information flow predicted by MLP is represented in Figure 4A: the model predicts no use of the reconstructed representations of the task, and the “exclusive” use of the comparison in the joint space does not require any cross-reference transformation. This phenomenon was clearly shown in unimodal, proprioceptive tasks involving only one arm (Tagliabue and McIntyre, 2011, 2013; Arnoux et al., 2017). The variance of the movement vector estimation corresponding to this optimal sensory processing is

(9) $$\sigma_{△}^{2}=2\,\sigma_{J}^{2}\;$$

#### Asymmetric Between-Arms Proprioceptive Tasks (aB-A_*P*_)

The asymmetric configuration of the limb during this test results in the impossibility to achieve the task by simply matching the joint signals from the two arms. Mathematically, this impossibility is represented by a large variance associated with the transformation of the proprioceptive joint signals between the left and right arm: $\sigma_{J_{l\to r}}^{2}=\sigma_{J_{r\to l}}^{2}\to$ ∞. The variances associated with the four concurrent target-effector comparisons are thus:

(10) $$\begin{array}{ccc} \sigma_{△\,J}^{2} & =\sigma_{J_{l}}^{2}+\sigma_{J_{l\to r}}^{2} & +\sigma_{J_{r}}^{2}+\sigma_{J_{r\to l}}^{2}\\ \sigma_{△\,E\,x\,J}^{2} & =\sigma_{J_{l}}^{2}+\sigma_{J_{l}\to E\,x\,J}^{2} & +\sigma_{J_{r}}^{2}+\sigma_{J_{r}\to E\,x\,J}^{2}\\ \sigma_{△\,R}^{2} & =\sigma_{J_{l}}^{2}+\sigma_{J_{l}\to R}^{2} & +\sigma_{J_{r}}^{2}+\sigma_{J_{r}\to R}^{2}\\ \sigma_{△\,E\,x\,R}^{2} & =\sigma_{J_{l}}^{2}+\sigma_{J_{l}\to R}^{2}+\sigma_{R\to E\,x\,R}^{2} & +\sigma_{J_{r}}^{2}+\sigma_{J_{r}\to R}^{2}+\sigma_{R\to E\,x\,R}^{2} \end{array}$$

If we assume that the cross-reference transformations from the left and right arm joints are characterized by the same variance ($\sigma_{J_{l}\to E\,x\,J}^{2}=\sigma_{J_{r}\to E\,x\,J}^{2}$ and $\sigma_{J_{l}\to R}^{2}=\sigma_{J_{r}\to R}^{2}$), the optimal sensory weighting predicted by MLP (Figure 4B), consists in encoding the position of the two hands perceived through proprioception in alternative reference frames, including the retinal one, rather than in joint space. This prediction is consistent with experimental observations on healthy subjects suggesting that retinal and external references contribute to the encoding of asymmetric between-arm tasks (Pouget et al., 2002; McGuire and Sabes, 2009; Jones and Henriques, 2010; Tagliabue and McIntyre, 2013; Arnoux et al., 2017). Tagliabue and McIntyre (2013) showed that it is the use of tasks that require asymmetric joint configurations in the above-mentioned studies that led to the visual reconstruction of proprioceptive signals.

The minimal achievable variability of the Δ estimation for these tasks is:

(11) $$\sigma_{△}^{2}=\sigma_{J_{r}}^{2}+\sigma_{J_{l}}^{2}+\frac{2\,\sigma_{J\to R}^{2}\,\sigma_{J\to E\,x\,J}^{2}}{\sigma_{J\to R}^{2}+\sigma_{J\to E\,x\,J}^{2}}$$

Thus in the aB-A_*P*_ tasks, the predicted variability of Δ is higher than for the W-A tasks, as experimentally observed (Tagliabue and McIntyre, 2013; Arnoux et al., 2017).

#### Symmetric Between-Arms Proprioceptive Tasks (sB-A_*P*_)

Experiments on healthy subjects have shown that, in contrast to what has been observed for the aB-A_*P*_ tests, the precision of this type of symmetric tasks is very similar to the one observed in within-arm tasks, W-A_*P*_, and no evidence of visual encoding was found (Arnoux et al., 2017). These similarities appear to be due to the same joint configuration of the arm holding the target and the arm performing the movement when achieving sB-A_*P*_ tasks. Hence, the movement can be controlled by a “direct” comparison between proprioceptive signals from homologous joints of the two limbs (Figure 4C).

The variances associated with the four concurrent target-effector comparisons for the mirroring tasks can be expressed as reported in Eq. 12.

(12) $$\begin{array}{cccc} \sigma_{△\,J}^{2} & =\sigma_{J_{l}}^{2}+\sigma_{J_{l\to r}}^{2} & +\sigma_{J_{r}}^{2}+\sigma_{J_{r\to l}}^{2} & \\ \sigma_{△\,E\,x\,J}^{2} & =\sigma_{J_{l}}^{2}+\sigma_{J_{l}\to E\,x\,J}^{2} & +\sigma_{J_{r}}^{2}+\sigma_{J_{r}\to E\,x\,J}^{2} & \\ \sigma_{△\,R}^{2} & =\sigma_{J_{l}}^{2}+\sigma_{J_{l}\to R}^{2} & +\sigma_{J_{r}}^{2}+\sigma_{J_{r}\to R}^{2}\;+\sigma_{R,M\,i\,r}^{2} & \\ \sigma_{△\,E\,x\,R}^{2} & =\sigma_{J_{l}}^{2}+\sigma_{J_{l}\to R}^{2}+\sigma_{R\to E\,x\,R}^{2} & +\sigma_{J_{r}}^{2}+\sigma_{J_{r}\to R}^{2}+\sigma_{R\to E\,x\,R}^{2} & \end{array}\;$$

These equations appear very similar to those describing the asymmetric between-arms tasks (Eq. 10), but there are two important differences, which reflect the different nature of the mirror task and the above-mentioned experimental observations. First, the parameter $\sigma_{R,M\,i\,r}^{2}$ is added to $\sigma_{△\,R}^{2}$. This parameter, which is very large ($\sigma_{R,M\,i\,r}^{2}\to$ ∞), reflects the impossibility to perform the task directly in retinal space: since the two hands must be in two distinct spatial locations, the task cannot be accomplished by matching the reconstructed image of the two hands on the retina. Second, the possibility of directly comparing proprioceptive signals from the two arms is represented by very low values of the variance associated to the transformation of the joint signals between the left and right arm: $\sigma_{J_{l\to r}}^{2}=\sigma_{J_{r\to l}}^{2}\to 0$. However, these parameters have not been removed from the equations to be able to describe the behavior of some of the stroke patients. An increase of the value of $\sigma_{J_{l\to r}}^{2}$ and $\sigma_{J_{r\to l}}^{2}$ can indeed be used to represent the observed difficulties of some patients in performing sB-A_*P*_ test with respect to the W-A_*P*_ tasks (Gurari et al., 2017).

Figure 4C reports the information flow predicted by MLP for two categories of patients: those that have difficulties in performing inter-hemispheric transformations ($\sigma_{J\,r↔l}^{2}$>0; dashed lines) and those that do not have this problem ($\sigma_{J_{r↔l}}^{2}\to 0$). For the latter category of patients, the proprioceptive information is encoded in joint space only, as for the within-arm tasks. For the patients with inter-hemispheric transformation issues MLP predicts an encoding of the information also in Extra-Joint and Extra-Retinal space.

Equation 13 reports the minimally achievable variability of the motor vector estimation.

$$\sigma_{△}^{2}=\sigma_{J_{r}}^{2}+\sigma_{J_{l}}^{2}+\frac{2\,\sigma_{J\,r↔l}^{2}\,\sigma_{J\to E\,x\,J}^{2}\,\left(\sigma_{J\to R}^{2}+\sigma_{R\to E\,x\,R}^{2}\right)}{\left(\sigma_{J\to R}^{2}+\sigma_{R\to E\,x\,R}^{2}\right)\,\left(\sigma_{J\to E\,x\,J}^{2}+\sigma_{J\,r↔l}^{2}\right)+\sigma_{J\to E\,x\,J}^{2}\,\sigma_{J\,r↔l}^{2}}$$

(13) $$\to \sigma_{J_{r}}^{2}+\sigma_{J_{l}}^{2}$$

In healthy subjects or in patients without inter-hemispheric transformation problems ($\sigma_{J_{r↔l}}^{2}\to 0)$, Figure 4C and Eq. 13 suggest that the sensory weighting and the motor vector variance tend to those predicted for the W-A_*P*_ tasks (Figure 4A and Eq. 9): encoding of the information in joint space only and minimal variability of the responses. This prediction is consistent with the experimentally observed similarities between the performances in sB-A_*P*_ and W-A_*P*_ tasks for healthy subjects (Tagliabue and McIntyre, 2013; Arnoux et al., 2017) and with the performances of some stroke patients (Herter et al., 2019).

The MLP prediction for stroke patients with a difficulty to compare joint signals from the affected to the less-affected side ($\sigma_{J\,r↔l}^{2}$>0) appears to provide some interesting insight into the patient’s deterioration of performances in the Mirror Position Test, with respect to the Within-arm Position Test (Gurari et al., 2017) discussed in section “Upper Limb Proprioceptive Deficits Post-stroke.” Equation 13 shows that the increased variability in the mirror task can be correctly predicted if the noise associated with the inter-hemispheric comparison of the joint signals ($\sigma_{J\,r↔l}^{2}$) is significantly larger than that for healthy patients. In other words, lower performances in patients assessed by the Mirror Position Test could be due to a problem in the neural inter-hemispheric processing and not due to a proprioceptive problem *per se*.

#### Cross-Modal Tasks (C-M_*P*_)

Contrary to the other categories of tasks, C-M_*P*_ tasks involve a visually memorized target which the patient has to match with the eyes closed (Figure 4D). In these tasks no direct comparison is possible between the target and effector. Thus, cross-reference transformations are strictly necessary. The variability associated with the four concurrent comparisons is:

(14) $$\begin{array}{ccc} \sigma_{△\,J}^{2} & =\sigma_{R}^{2}+\sigma_{R\to J}^{2} & +\sigma_{J}^{2}\\ \sigma_{△\,E\,x\,J}^{2} & =\sigma_{R}^{2}+\sigma_{R\to J}^{2}+\sigma_{J\to E\,x\,J}^{2} & +\sigma_{J}^{2}+\sigma_{J\to E\,x\,J}^{2}\\ \sigma_{△\,R}^{2} & =\sigma_{R}^{2} & +\sigma_{J}^{2}+\sigma_{J\to R}^{2}\\ \sigma_{△\,E\,x\,R}^{2} & =\sigma_{R}^{2}+\sigma_{R\to E\,x\,R}^{2} & +\sigma_{J}^{2}+\sigma_{J\to R}^{2}+\sigma_{R\to E\,x\,R}^{2} \end{array}$$

$\sigma_{R}^{2}$ refers to the variability associated with the retinal inputs of the target location. If we assume that the noise associated with the transformation of the sensory signals from retinal to joint space and from joint to retinal space are similar ($\sigma_{R\to J}^{2}=\sigma_{J\to R}^{2}=\sigma_{J↔R}^{2}),$ then the sensory weights predicted by the MLP are those represented in Figure 4D and the corresponding minimal variance of the estimated movement vector Δ is:

(15) $$\sigma_{△}^{2}=\sigma_{J}^{2}+\sigma_{R}^{2}+\frac{\sigma_{J↔R}^{2}}{2}$$

It follows that degraded performances of stroke patients when performing this category of tasks could be due, not only to a noisy proprioceptive system, but also to difficulties in the encoding of retinal information in joint space or, vice-versa, proprioceptive information in a retinal reference.

### Application of the Optimal Sensory Integration Theory to Visual Compensation Tests

The MLP also renders predictions for the visual compensation tests in which stroke patients can use visual feedback to perform the tasks. In the following we will apply the Concurrent Model to the execution of the same four categories of tasks analyzed in the previous section (W-A, aB-A, sB-A, and C-M) but including the availability of visual information about both target and effector position. $\sigma_{R}^{2}$ will be used to refer to the variability associated with the retinal inputs of both target and effector locations.

#### Within-Arm Visuo-Proprioceptive Tasks (W-A_*VP*_)

Equations 16 represent the variance associated with the four concurrent comparisons for the within-arm tasks using both proprioceptive and visual information.

(16) $$\begin{array}{ccc} \sigma_{△\,J}^{2} & =\sigma_{J}^{2} & +\sigma_{J}^{2}\\ \sigma_{△\,E\,x\,J}^{2} & =\sigma_{J}^{2}+\sigma_{J\to E\,x\,J}^{2} & +\sigma_{J}^{2}+\sigma_{J\to E\,x\,J}^{2}\\ \sigma_{△\,R}^{2} & =\sigma_{R}^{2} & +\sigma_{R}^{2}\\ \sigma_{△\,E\,x\,R}^{2} & =\sigma_{R}^{2}+\sigma_{R\to E\,x\,R}^{2} & +\sigma_{R}^{2}+\sigma_{R\to E\,x\,R}^{2} \end{array}$$

The first two equations, representing the proprioceptive comparison in Joint and Extra-Joint space, are identical to those reported for the W-A_*P*_ tasks in Eq. 8. The last two equations represent the visual comparison in Retinal and Extra-Retinal space. The target and effector images on the retina can be compared directly. Therefore, the variability of the retinal comparison, $\sigma_{△\,R}^{2}$, simply corresponds to the sum of the variability of the retinal information about the target and the effector position. The visual extra-retinal comparison, △*ExR*, on the other hand, must include the terms $\sigma_{R\to E\,x\,R}^{2}$, associated with the transformation from the retinal to the extra-retinal reference frame.

**FIGURE 5:**
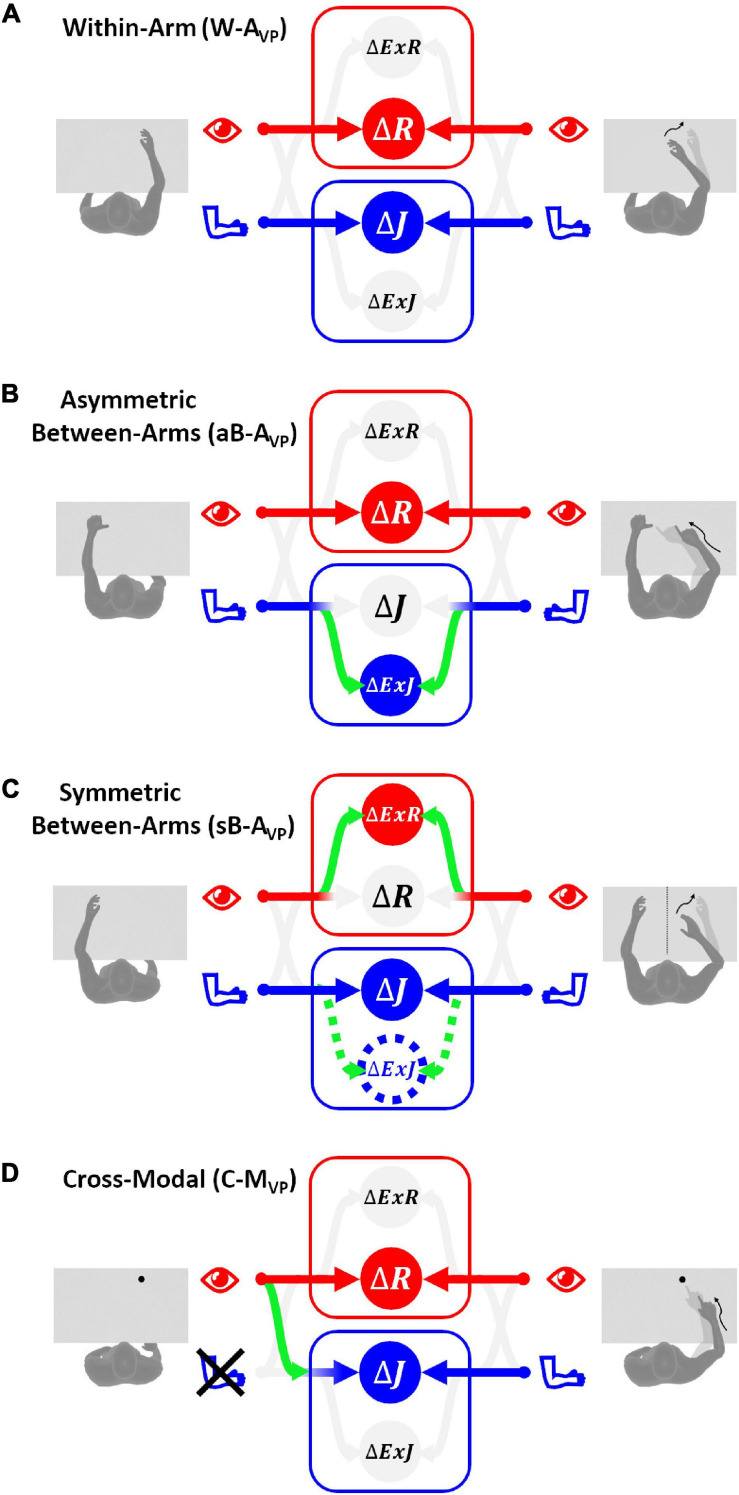
Sensory information flow predicted for visual compensation tests. The model results are reported separately for the four categories of tests with vision: **(A)** within-arm task (W-A_*VP*_), both proprioceptive and visual signals from the target and the effector can be directly compared in their primary reference frames. **(B)** Asymmetric between-arms task (aB-A_*VP*_), the proprioceptive target-effector comparison cannot be encoded in the primary joint space, while the visual comparison can be performed directly in the retinal space. **(C)** Symmetric between-arms task (sB-A_*VP*_), the proprioceptive target-effector comparison is performed directly in the primary joint space only for patients without inter-hemispheric transformation deficits. In order to compare the visual position of the two hands that are far apart, all patients have to encode the retinal signals in some extra-retinal space. **(D)** Cross-modal task (C-M_*VP*_), the proprioceptive target-effector comparison in joint space can be performed through a cross-reference transformation, while visual signals from the target and the reaching movement can be directly compared in retinal space.

As shown in Figure 5A, MLP predicts that for the W-A_*VP*_ task there would be no sensory encoding in extra-joint or extra-retinal reference. This is due to the fact that, for both visual and proprioceptive modality, the information can be directly compared in the reference frame corresponding to the originating sensory system. The retinal and joint comparisons are weighted as predicted by the standard MLP formulation (Eq. 3), taking into account only the relative variance of the available sources of information (Ernst and Banks, 2002). The variability of the estimation of the movement vector Δ corresponding to this optimal sensory weighting is:

(17) $$\sigma_{△}^{2}=\frac{2\,\sigma_{J}^{2}\,\sigma_{R}^{2}}{\sigma_{J}^{2}+\sigma_{R}^{2}}$$

The comparison of these results with the corresponding prediction for the proprioceptive task (Eq. 9) suggests that patients should be able to visually compensate in this category of tasks, independently from their ability to perform cross-reference transformations: $\frac{2\,\sigma_{J}^{2}\,\sigma_{R}^{2}}{\sigma_{J}^{2}+\sigma_{R}^{2}}$ is always smaller than $2\,\sigma_{J}^{2}$ and this difference is not affected by the variance of the sensory transformations reported in Eq. 16. This comparison also shows that, the stronger the proprioceptive deficit, the larger will be the advantage provided by using visual information. This prediction is consistent with the observation that stroke patients can compensate through vision for their proprioceptive deficits in this type of tasks (Torre et al., 2013).

#### Asymmetric Between-Arms Visuo-Proprioceptive Tasks (aB-A_*VP*_)

In these tasks, as previously explained for the aB-A_*P*_ tests, a direct comparison between the proprioceptive information in joint space is not possible ($\sigma_{J_{l↔r}}^{2}\to \infty$). On the other hand, target and effector can be compared directly in retinal coordinates because the task achievement corresponds to the matching of their respective positions on the retina. As a consequence, the concurrent comparison for these tasks are associated with the following variances:

(18) $$\begin{array}{ccc} \sigma_{△\,J}^{2} & =\sigma_{J_{l}}^{2}+\sigma_{J_{l\to r}}^{2} & +\sigma_{J_{r}}^{2}+\sigma_{J_{r\to l}}^{2}\\ \sigma_{△\,E\,x\,J}^{2} & =\sigma_{J_{l}}^{2}+\sigma_{J_{l}\to E\,x\,J}^{2} & +\sigma_{J_{r}}^{2}+\sigma_{J_{r}\to E\,x\,J}^{2}\\ \sigma_{△\,R}^{2} & =\sigma_{R}^{2} & +\sigma_{R}^{2}\\ \sigma_{△\,E\,x\,R}^{2} & =\sigma_{R}^{2}+\sigma_{R\to E\,x\,R}^{2} & +\sigma_{R}^{2}+\sigma_{R\to E\,x\,R}^{2} \end{array}$$

Figure 5B shows that the sensory information flow corresponding to the minimal variability of the aB-A_*VP*_ task consists, theoretically, in the encoding of proprioceptive information in extra-joint spaces, while visual information is directly encoded in retinal space. Proprioceptive information is not encoded in the joint reference frame, because, as discussed for the corresponding proprioceptive task aB-A_*P*_, the comparison in the joint space is not possible. The visual information is not encoded in extra-retinal references, because, although △*ExR* would be theoretically possible, it would fully covary with △*R*. In other words, the extra-retinal encoding would not provide any additional information over the retinal encoding, and would not contribute to reduce the variance of the motor vector estimate, which is given in Eq. 19.

(19) $$\sigma_{△}^{2}\to \frac{2\,\sigma_{R}^{2}\,\left(\sigma_{J_{r}}^{2}+\sigma_{J_{l}}^{2}+2\,\sigma_{J\to E\,x\,J}^{2}\right)}{\sigma_{J_{r}}^{2}+\sigma_{J_{l}}^{2}+2\,\sigma_{J\to E\,x\,J}^{2}+2\,\sigma_{R}^{2}}$$

The comparison of this result with the one obtained in Eq. 11 for the corresponding proprioceptive task aB-Ap (see the Supplementary Section 4), shows that in normal conditions the noisiness of the motor vector estimation in the visuo-proprioceptive task is always smaller than for the proprioceptive task. Thus, MLP predicts for this kind of asymmetric tasks that the patients should be able to compensate their proprioceptive deficits by using vision, consistent with experimental observations (Scalha et al., 2011).

#### Symmetric Between-Arms Visuo-Proprioceptive Tasks (sB-A_*VP*_)

For these tasks, the considerations about inter-hemispheric transfer of joint signals presented for the corresponding proprioceptive tasks (sB-A_*P*_) remain valid: the value of the $\sigma_{J_{r↔l}}^{2}$ parameter allows distinguishing patients with problems in comparing joint information from the two arms ($\sigma_{J_{r↔l}}^{2}>0$) from healthy subjects and patients not showing this deficit ($\sigma_{J_{r↔l}}^{2}\to 0$). The considerations about the impossibility of performing the task by directly comparing the visual feedback about the target and the effector ($\sigma_{R,M\,i\,r}^{2}\to \infty$) also remain valid.

Equations 20, which describe the variability associated with the four concurrent comparisons for this type of tasks, differ from the analogous equations of the proprioceptive sB-A_*P*_ task (Eq. 12), simply by the fact that △*R* and △*ExR* are computed from the available retinal information (*R*) and not through cross-reference transformations of proprioceptive signals (*J*→*R*).

(20) $$\begin{array}{cccc} \sigma_{△\,J}^{2} & =\sigma_{J_{l}}^{2}+\sigma_{J_{l\to r}}^{2} & +\sigma_{J_{r}}^{2}+\sigma_{J_{r\to l}}^{2} & \\ \sigma_{△\,E\,x\,J}^{2} & =\sigma_{J_{l}}^{2}+\sigma_{J_{l}\to E\,x\,J}^{2} & +\sigma_{J_{r}}^{2}+\sigma_{J_{r}\to E\,x\,J}^{2} & \\ \sigma_{△\,R}^{2} & =\sigma_{R}^{2} & +\sigma_{R}^{2} & +\sigma_{R,M\,i\,r}^{2}\\ \sigma_{△\,E\,x\,R}^{2} & =\sigma_{R}^{2}+\sigma_{R\to E\,x\,R}^{2} & +\sigma_{R}^{2}+\sigma_{R\to E\,x\,R}^{2} & \end{array}$$

The optimal weights associated with the four concurrent target-response comparisons are represented in Figure 5C. The predicted sensory information flow is reported for patients both with and without inter-hemispheric transformation deficits. The MLP prediction suggests that to achieve optimal performance stroke patients with problems in comparing joint signals from the two arms should encode proprioceptive information in both joint and extra-joint space, and visual information in extra-retinal space only. Patients without inter-hemispheric communication issues, on the other hand, should encode proprioceptive information in joint space only and visual information in extra-retinal references only.

The variability of the optimal motor vector estimation is shown in Eq. 21. The equation reports, first, the prediction for patients with inter-hemispheric transformation deficits ($\sigma_{J_{r↔l}}^{2}>0$) and then for patients without problems in comparing the sensory information coming from the two arms ($\sigma_{J_{r↔l}}^{2}\to 0$).

$$\sigma_{△}^{2}\to \frac{\begin{array}{c} 2\,\left(\sigma_{R}^{2}+\sigma_{R\to E\,x\,R}^{2}\right)\,\left(\left(\sigma_{J_{r}}^{2}+\sigma_{J_{l}}^{2}\right)\,\left(\sigma_{J\to E\,x\,J}^{2}+\sigma_{J\,r↔l}^{2}\right)+2\,\sigma_{J\to E\,x\,J}^{2}\,\sigma_{J\,r↔l}^{2}\right) \end{array}}{\left(\sigma_{J\to E\,x\,J}^{2}+\sigma_{J\,r↔l}^{2}\right)(\begin{array}{c} \sigma_{J_{r}}^{2}+\sigma_{J_{l}}^{2}+2\sigma_{R}^{2}+2\sigma_{R\to E\,x\,R}^{2})+2\sigma {}^{2}{}_{J\to E\,x\,J}\sigma {}^{2}{}_{J\,r↔l} \end{array}}$$

(21) $$\to \frac{2\,\left(\sigma_{R}^{2}+\sigma_{R\to E\,x\,R}^{2}\right)\,\left(\sigma_{J_{r}}^{2}+\sigma_{J_{l}}^{2}\right)}{\left(2\,\sigma_{R}^{2}+2\,\sigma_{R\to E\,x\,R}^{2}\right)+\left(\sigma_{J_{r}}^{2}+\sigma_{J_{l}}^{2}\right)}$$

The comparison of these results with those reported in Eq. 13 for the corresponding proprioceptive task, sB-A_*P*_ (see Supplementary Section 4 for details) suggests different visual compensation mechanism for the patient with and without inter-hemispheric transformation issues. For patients without problems in comparing joint signals from the two arms, the availability of visual information should result in a direct reduction of the noisiness of the estimation of the motor vector. For the patients with problems in comparing joint information from the two arms, the possibility to reduce the noise of the motor vector estimate appears to be more limited and to depend on the relative noisiness associated to cross-reference transformations. The inability observed in some stroke patients to use visual information to improve their performances with respect to analogous proprioceptive tasks (Semrau et al., 2018; Herter et al., 2019) could, therefore, be due to difficulties in performing inter-hemispheric and cross-reference transformations.

#### Cross-Modal Tasks (C-M_*VP*_)

As shown in Figure 5D, since the target is not perceived proprioceptively, no direct comparison is possible between the target and effector in joint space in this task. Hence a cross-reference transformation ($\sigma_{R\to J}^{2}$) would be necessary to make use of the proprioceptive signal on effector position. The variability associated with the four concurrent comparisons is given in Eq. 22.

(22) $$\begin{array}{ccc} \sigma_{△\,J}^{2} & =\sigma_{R}^{2}+\sigma_{R\to J}^{2} & +\sigma_{J}^{2}\\ \sigma_{△\,E\,x\,J}^{2} & =\sigma_{R}^{2}+\sigma_{R\to J}^{2}+\sigma_{J\to E\,x\,J}^{2} & +\sigma_{J}^{2}+\sigma_{J\to E\,x\,J}^{2}\\ \sigma_{△\,R}^{2} & =\sigma_{R}^{2} & +\sigma_{R}^{2}\\ \sigma_{△\,E\,x\,R}^{2} & =\sigma_{R}^{2}+\sigma_{R\to E\,x\,R}^{2} & +\sigma_{R}^{2}+\sigma_{R\to E\,x\,R}^{2} \end{array}$$

MLP predicts that the optimal solution for this type of tasks is to encode the proprioceptive and visual information directly in joint and retinal space, respectively. The variance of the estimated movement vector corresponding to this optimal solution is given in Eq. 23.

(23) $$\sigma_{△}^{2}=\frac{\sigma_{R}^{2}\,\left(2\,\sigma_{J}^{2}+\sigma_{R}^{2}+2\,\sigma_{R\to J}^{2}\right)}{\sigma_{J}^{2}+\sigma_{R\to J}^{2}+\sigma_{R}^{2}}$$

The comparison between this result and the variability of the movement vector estimation in the corresponding proprioceptive task C-M_*P*_ of Eq. 15 (see Supplementary Section 4) shows that, unless visual information is extremely noisy, its availability should lead to a reduction of the variance of △. It follows that, for this category of task, MLP predicts that the patients should show a clear visual compensation of their proprioceptive deficit. This prediction is in agreement with the visual compensation experimentally observed in stroke patients for this category of tasks (Darling et al., 2008; Scalha et al., 2011).

## Reinterpretation of Experimental Observations About Proprioceptive Deficits and Visual Compensation

After having described the theoretical sensory information flow underlying the four categories of tasks used to test proprioception and visual compensation, we assess the ability of the model to capture the relevant experimental findings described in the first sections. In order to avoid data overfitting, the number of model parameters is reduced to six: the noise of the joint ($\sigma_{J}^{2}$) and retinal ($\sigma_{R}^{2}$) signals and the noise associated to sensory transformations ($\sigma_{T}^{2}$) in healthy subjects; for patients, three terms representing the noise added to the joint signal of the more affected (*N*_*J_m_*_) and less affected arm (*N*_*J_l_*_) and to the sensory transformations (*N_T_*) due to the deficit of stroke patients.

For this analysis, we will consider three distinct type of patients: P, with proprioceptive deficits only (*N*_*J_m_*_ and *N*_*J*_*l*__ > 0 and *N*_*T*_ = 0); C, with cross-reference processing deficits only (*N*_*J*_*m*_*NJ*_*l*__ = 0 and *N*_*T*_ > 0); and P+C, with combined proprioceptive and cross-reference processing deficits (*N*_*J_m_*_, *N*_*J_l_*_ and *N*_*T*_ > 0). In patients of type P, only the noisiness of the proprioceptive joint signals $\sigma_{J}^{2}$ is increased with respect to healthy subjects. For patients of type C, only the noise associated to the sensory transformation ($\sigma_{R↔J}^{2}$, $\sigma_{R\to E\,x\,R}^{2}$, $\sigma_{J\to E\,x\,J}^{2}$, $\sigma_{J\,r↔l}^{2}$) is increased with respect to healthy subjects. For patients of type P+C the noise is increased for both proprioception and transformations.

**FIGURE 6:**
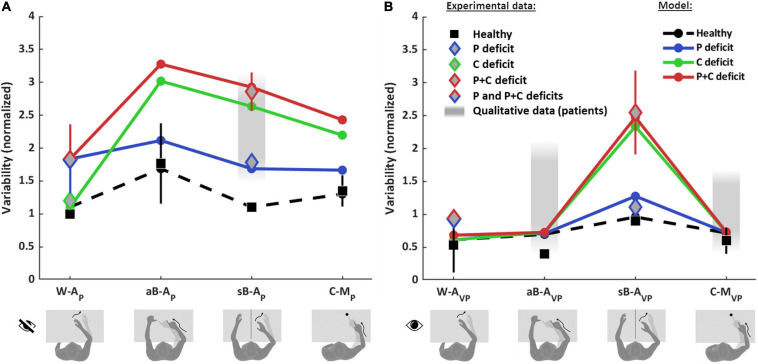
Model predictions and experimental observations for proprioception and visual compensation tests. Data and predictions are reported for the three types of patients: purely proprioceptive deficit (P), cross-reference deficit (C) and mixed proprioceptive and cross reference deficit (P+C), and for healthy subjects. All values are normalized with respect to the variability of healthy subjects in the within-arm proprioceptive task (W-A_*P*_). If more than one quantitative study was included in the analysis for a particular task and group of subjects, the mean and standard deviation (vertical whiskers) were used to represent experimental data. Qualitative data from stroke patients (gray filled rectangles) were not used for the fitting. **(A)** Proprioceptive tests. For the W-A_*P*_ tasks, the mean of healthy subjects’ data is used as reference value for the normalizations. For this tasks, C patients’ data can be distinguished from P and P+C patients. For the aB-A_*P*_ tasks, only data from healthy subject could be included. For the sB-A_*P*_ tasks, both healthy subjects and stroke patients data are available: patients with P deficits perform better and could hence be distinguished from P+C patients. The model results suggest that the data associated to the P+C patients is similar to what is expected also for C patients. The reported qualitative results refer to the same C patients of the W-A_*P*_ task. For the C-M_*P*_ task, only results from healthy subjects were included. **(B)** Visual compensation tests. For the W-A_*VP*_ tasks, data from healthy subjects and from stroke patients are reported. For the aB-A_*VP*_ task, quantitative data were included for healthy subjects. For patients only qualitative observations were found. For the sB-A_*VP*_ tasks, data from healthy subjects, P patients and P+C patients are reported. The model results suggest that the experimental data associated to P+C patients correspond also to the results expected for C patients. For the C-M_*VP*_ tasks, as for the asymmetric tasks, quantitative data were found for healthy subjects, but only qualitative observations for patients. Full details about the studies from which the data have been retrieved are reported in Supplementary Tables 1–4.

Figure 6 shows the comparison between the quantitative experimental data found in the literature and the prediction of the MLP model for the four categories of proprioceptive tasks (Figure 6A) and for the same four tasks performed using vision to compensate for proprioceptive deficits (Figure 6B). In order to be able to apply the model to the whole dataset, the results from different studies have to be comparable. This was achieved through their normalization with respect to a reference task. To be able to perform the normalization, among the numerous studies that can be found in the literature, only those reporting a quantitative comparison between at least two of the four categories of tasks (W-A, sB-A, aB-A, and C-M) could be included in the dataset. Performance data of healthy subjects were retrieved from Van Beers et al. (1996), Ernst and Banks (2002), Butler et al. (2004), Monaco et al. (2010), Tagliabue and McIntyre (2011), Torre et al. (2013), Khanafer and Cressman (2014), Cameron and López-Moliner (2015), Arnoux et al. (2017), Herter et al. (2019), and Marini et al. (2019) and those of stroke patients from Scalha et al. (2011), Torre et al. (2013), Dos Santos et al. (2015), Contu et al. (2017), Gurari et al. (2017), Rinderknecht et al. (2018), Herter et al. (2019), and Ingemanson et al. (2019). Details about the dataset, the fitting algorithm and the quantification of the obtained results are given in Supplementary Section 5.

Figure 6 shows that the model predicts very different results for healthy subjects and for the three type of patients (P, C, and P+C), depending on the considered task.

For healthy subjects, MLP reproduces well the experimentally observed modulations of the precision among the eight tasks. In particular, the model correctly predicts that the asymmetric test (aB-A_*P*_) is the least precise (largest variability) among the proprioceptive tasks (Figure 6A) and that the symmetric test (sB-A_*VP*_) is the less precise among the tasks using vision (Figure 6B).

For stroke patients, the results of Figure 6A show that the model seems to capture the different experimental data for the within-arm tasks (W-A_*P*_), suggesting that the heterogeneity of the results would be partially explained by differentiating C patients (Gurari et al., 2017) from P and P+C patients (Dos Santos et al., 2015; Contu et al., 2017; Rinderknecht et al., 2018). For the asymmetric tasks (aB-A_*P*_), the model predicts a very high variability for the C and P+C patients while the increase with respect to the W-A_*P*_ task is moderate for P patients. We do not have, however, experimental data to validate the predictions for the patients in this task. For the sB-A_*P*_ task, the model well captures the heterogeneity of the patients’ dataset by distinguishing P patients (Herter et al., 2019) from C and P+C patients (Herter et al., 2019; Ingemanson et al., 2019). Interestingly, this classification is consistent with the fact that P patients were able to visually compensate in the sB-A_*VP*_ task, whereas C and P+C patients were not able to compensate (Herter et al., 2019). The experimental data represented by a red diamond for sB-A_*P*_ task were associated with to P+C patients in the fitting procedure, because observations in the literature suggest that P+C patients are more common than C patients. The model prediction suggests, however, that these data could also include C patients. The prediction for the sB-A_*P*_ task is also consistent with qualitative observations of Gurari et al. (2017) that the same patients that performed without difficulties the W-A_*P*_ task (classified as C patients) showed significant deficits in a symmetric task. For the cross-modal tasks (C-M_*P*_), the model predicts that performances of C and P+C patients would be characterized by a variability significantly larger than that of P patients, similarly to the sB-A_*P*_ task.

Concerning the patients’ ability to visually compensate for their proprioceptive deficits (Figure 6B), the model predicts that in the W-A_*VP*_ task all three types of patients (P, C and P+C) should be able to use visual information to improve performance to that of healthy subjects. This prediction is consistent with the experimental observation of Torre et al. (2013) that stroke patients can fully compensate with vision when performing this kind of task, where the information about the target and the effector could be compared directly in both joint and retinal space. For the aB-A_*VP*_ tasks, the model predicts the same full visual compensation as for the within-arm task. Although we could not find any quantitative experimental results for patients in this type of tasks, the model prediction is coherent with the qualitative observation of Scalha et al. (2011) that patients can significantly improve their performances with vision. For the sB-A_*VP*_ tasks, the model prediction is very different from the other tasks and it matches the different results obtained by Herter et al. (2019) for patients with low and high levels of visual compensation. The model predictions for this task suggests that the group of patients showing low visual compensation (higher variability) could confound C and P+C patients, although the patients with the ability to visually compensate (lower variability) are probably of type P. For this task, as for the corresponding proprioceptive test sB-A_*P*_, the model prediction suggests that the experimental data point represented by a red diamond could confound C and P+C patients. For the C-M_*VP*_ tasks, the same considerations apply as for the aB-A_*VP*_ task, in terms of model predictions and of matching with qualitative observations.

Altogether, these results suggest that only the W-A_*P*_ tasks can be considered as “pure proprioception tests.” This expression here refers to those tests whose outcome is affected only by deficits of the proprioceptive system, and not by other factors, such as the inability to perform sensory transformations. In contrast, sB-A_*P*_, aB-A_*P*_, and C-M_*P*_ tasks appear to confound proprioceptive deficits and cross-reference transformation deficits, since they are affected by P, C, or P+C deficits. These results also suggest that the visual compensation tests for sB-A_*VP*_ tasks can assess the patients’ ability to perform cross-reference transformations. The reinterpretation of the data of the literature through the MLP framework represented in Figure 6 additionally suggests that most of the tested stroke patients have mixed P+C deficits (Dos Santos et al., 2015; Contu et al., 2017; Rinderknecht et al., 2018; Herter et al., 2019), but that there are also clear examples of C (Gurari et al., 2017) and P (Herter et al., 2019) categories of patients.

In conclusion, the proposed stratification of patients presented here based on their deficits (P, C, and P+C) appears to be able to explain, and at least partially reconciliate, the different outcomes experimentally obtained with various assessments currently in use in clinical research.

## Insights From Brain Lesions and Functional Anatomy Studies

The neural network responsible for proprioceptive processing seems widely distributed over cortical and subcortical structures (Ben-Shabat et al., 2015; Kessner et al., 2016; Semrau et al., 2018). Beyond the integrity of S1, with a clear impact on proprioception, neural correlates of proprioceptive deficits after stroke remain incompletely understood (Ingemanson et al., 2019). Moreover, no study has yet been undertaken to stratify stroke patients according to the categorization of deficits described in the previous section. However, to probe the clinical potential of this approach we present here a short non-systematic review on brain structures involved in either “pure” proprioceptive perception or cross-reference processing. To that end, we reviewed studies that used functional imaging (fMRI, PET, and EEG after a non-systematic PubMed screening) during proprioceptive and visuo-proprioceptive tasks, as well as imaging-based lesion-symptom mapping (LSM) studies. This should provide a first approximative view on whether brain areas may potentially be dissociated as a function of their involvement in proprioceptive processing according to the described task affordances.

However, there is a caveat: as discussed in the previous section, most stroke patients likely have mixed deficits affecting both proprioception and cross-reference processing. Since a mixed deficit would alter the patients’ performances in all task categories (Figure 6), only a dedicated protocol would allow dissociating the structures specifically involved in tasks requiring cross-reference processing or not. Unsurprisingly, cortical networks seemed to overlap to a large extent among the reviewed articles, and proprioceptive test categorization did not provide a clear dissociation between the cortical areas activated during tests belonging to one or the other category.

In addition to S1, a number of regions within the posterior parietal cortex (PPC) were identified as critical for proprioceptive perception, assessed with either a W-A_*P*_ (Rinderknecht et al., 2018; Kessner et al., 2019) task or a sB-A_*P*_ task (Dukelow et al., 2010; Findlater et al., 2016, 2018; Meyer et al., 2016). But the lack of between-task comparisons does not allow for a distinction between the lesions sites affecting primarily the proprioceptive sense *per se* or cross-reference processing. Furthermore, based on these results we cannot conclude whether hemispheric dominance may be related to either proprioception or cross-reference processing.

A comparative approach with different types of tasks is needed to elucidate the sensory deficit and to eventually associate a given sensory deficit to particular brain regions. Unless the study assesses and compares different tasks (Semrau et al., 2018; Herter et al., 2019), or uses functional imaging (Van de Winckel et al., 2012; Ben-Shabat et al., 2015), we cannot draw clear conclusions on which brain areas are important for either sensory function.

According to the presented MLP predictions, we consider W-A_*P*_ assessments to be “purely” proprioceptive (Figure 4A) in contrast to assessments which involve cross-reference processing (aB-A_*P*_, sB-A_*P*_, C-M_*P*_: see Figures 4B–D). We therefore attempted to classify the reviewed functional brain imaging studies accordingly and to probe whether this categorization might result in a processing-specific topological cerebral organization.

“Pure” proprioceptive processing, assessed with a W-A_*P*_ tasks seemed to entail primarily the activation of M1 and S1 (Butler et al., 2004; Marini et al., 2019). W-A_*P*_ (Figure 4A) tasks, the simplest tasks in terms of computational load (see section “Application of the Optimal Sensory Integration Theory to Proprioception Assessment Tests”), are presumably based on simpler networks. In contrast, the mirror task (a sB-A_*P*_ task) seem to involve cross-reference processing. And fMRI revealed that a larger brain network was involved compared to W-A_*P*_ tasks, with higher activation of the supramarginal gyrus (SMG) and superior temporal gyrus (STG) (Iandolo et al., 2018), in line with Ben-Shabat et al. (2015). In theory, the same mirror task with visual feedback also involves cross-reference processing (sB-A_*VP*_: Figure 5C). An LSM study showed that patients with lesions to the SMG did not improve their performance when adding visual feedback in the mirror test (sB-A_*P*_ vs. sB-A_*VP*_), a result presumably related to cross-reference processing deficit (Semrau et al., 2018). Patients that improved to normal performance with vision, i.e., presumably patients with “pure” proprioceptive deficit (Figure 6), had smaller lesions that primarily affected white-matter tracts carrying proprioceptive information rather than lesions in parietal association areas (Semrau et al., 2018). This result is therefore consistent with a specific role of the parietal association areas in cross-reference processing.

Other proprioceptive tasks such as aB-A_*P*_ and C-M_*P*_, known for the visual encoding of proprioceptive information requiring cross-reference transformations, have also been associated to parietal activation. Pellijeff et al. (2006) showed that the fMRI response was specifically enhanced in the superior parietal lobule (SPL) and Precuneus (medial part of the PPC) in a thumb and chin pointing task requiring an update of the limb posture to achieve the task. Similarly, using PET, Butler et al. (2004) showed a greater activity in the SPL in the C-M_*P*_ reaching task. Within the PPC, Grefkes et al. (2002) showed that the activity in the anterior intraparietal sulcus (IPS) was specifically enhanced during tactile object recognition. This task, requiring cross-modal visuo-tactile information transfer, involved the anterior IPS in stroke patients (Van de Winckel et al., 2012).

**FIGURE 7:**
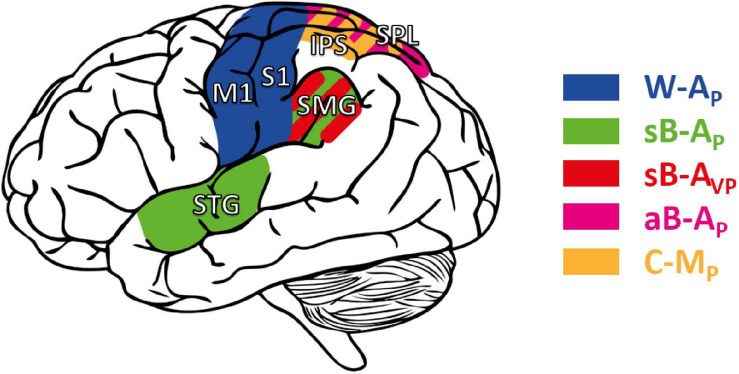
Cortical areas potentially involved in proprioceptive and cross-reference processing. M1, S1: primary motor and somatosensory area, respectively. STG: superior temporal gyrus. Posterior parietal cortex (PPC), including the supramarginal gyrus (SMG), the superior parietal lobule (SPL), and the intraparietal sulcus (IPS). Areas in blue: cortical areas preferentially involved in W-A_*P*_, purely proprioceptive tasks (Butler et al., 2004; Iandolo et al., 2018; Marini et al., 2019). Green: enhanced activity when the task requires cross-reference processing in symmetric between-arms tasks (sB-A_*P*_) as in Ben-Shabat et al. (2015) and Iandolo et al. (2018); red: in the symmetric between-arms tasks (sB-A_*VP*_) as in Semrau et al. (2018); purple: in the asymmetric between-arms task (aB-A_*P*_) as in Pellijeff et al. (2006); yellow: in cross-modal (C-M_*P*_) tasks as in Grefkes et al. (2002), Butler et al. (2004), and Van de Winckel et al. (2012).

Overall, these studies tended to show that “pure” proprioceptive processing involves mainly S1, whereas cross-reference processing recruits specifically the parietal associative cortex. Figure 7 shows the main trends for task-specific involvement that might be read out as: (i) Tasks excluding visual inputs and that do not require cross-reference processing (W-A_*P*_) showed a trend for activating preferentially anterior parietal areas (M1, S1). (ii) Tasks excluding visual inputs but requiring cross-reference processing (sB-A_*P*_), or for which visual processing requires cross-reference transformations (sB-A_*VP*_), seemed to entail additional activation of superior temporal and inferior-lateral PPC areas. (iii) Tasks that impose cross-modal processing, for which a visual encoding of the proprioceptive information has been reported in healthy subjects (aB-A_*P*_, C-M_*P*_: Tagliabue and McIntyre, 2011, 2013), tended to activate the superior-medial PPC areas. There might thus be a gradient within PPC from inferior-lateral to more superior-medial activation with increasing cross-reference processing demands.

## Discussion

Here, we present a reinterpretation of proprioceptive post-stroke deficits affecting manual control, and of the ability of patients to compensate for these deficits using vision. This theoretical analysis uses the MLP (Ernst and Banks, 2002) and a new formulation of the “Concurrent Model” for multi-sensory integration (Tagliabue and McIntyre, 2011, 2014). The rationale for this work hinges on the conceptual approach that the sensory space in which the information is encoded is not limited to the sensory system from which the signal originates. This concept is supported by evidence that retinal encoding of purely proprioceptive task-contingent stimuli (i.e., in absence of vision) occurs in some pointing tasks (Pouget et al., 2002; Sarlegna and Sainburg, 2007; McGuire and Sabes, 2009; Jones and Henriques, 2010; Tagliabue and McIntyre, 2013; Arnoux et al., 2017). Hence, it is questionable whether some tasks, traditionally classified as being proprioceptive, can be considered as relying on proprioceptive processing only. Moreover, there is evidence that the efficacy of visual compensation is task-dependent (Scalha et al., 2011; Torre et al., 2013; Semrau et al., 2018; Herter et al., 2019). Therefore, it is also questionable whether different visual compensation tasks imply similar sensory processing.

### A Useful Categorization of Proprioceptive Assessments

Applying this concept to clinical proprioceptive deficits and visual compensation tests, we attempt to dissociate purely proprioceptive deficits from those affecting cross-reference processing. We were able to show that tasks described as proprioceptive in clinical practice are likely to involve cross-reference processing. As a consequence, task performances in patients may not specifically depend on a strictly proprioceptive deficit, but may also depend on deficits in performing cross-reference transformations. Clinical and nonclinical methods as well as tasks that assess proprioceptive function and visual compensation have been reviewed and compared through this new conceptual framework. This process led to a new classification of methods for proprioceptive assessments into four categories, which differ by the requirement of performing a task by encoding the information directly in the reference frame associated with sensory receptors: proprioceptive (joint) space and visual (retinal) spaces, respectively. In the first category both visual and proprioceptive information can be encoded in the primary sensory space. The second category includes those tasks in which visual, but not proprioceptive, information can be encoded in the primary sensory space. In the tasks of the third category, proprioceptive, but not visual, information can be encoded in the primary sensory space. The tasks of the fourth category require encoding in non-primary sensory space for both proprioception and vision.

The present analysis suggests that only assessments using a within-arm task represent a “pure” proprioceptive test, because their execution does not require any cross-reference transformation of proprioceptive information. On the contrary, tasks including a between-arms condition, and in particular those that are asymmetric with respect to the body-midline, likely require cross-reference transformations, among which a reconstruction of the task in visual space. As a consequence, these tests do not specifically assess proprioceptive integrity *per se*, but also the ability to perform sensory transformations. Lesion-symptom and functional imaging studies support this hypothesis (Grefkes et al., 2002; Butler et al., 2004; Pellijeff et al., 2006; Van de Winckel et al., 2012; Ben-Shabat et al., 2015; Iandolo et al., 2018; Semrau et al., 2018). The neural network involved in between-arms tasks is wider compared to the network involved in simpler, within-arm, proprioceptive tasks (Ben-Shabat et al., 2015; Iandolo et al., 2018) and includes the PPC which is known to be involved in cross-modal processing (Grefkes et al., 2002; Yau et al., 2015). Moreover, the use of visual information in between-arms mirror (symmetric) tasks might be dependent on the ability to perform cross-reference transformations (Semrau et al., 2018; Herter et al., 2019). Hence, the common practice in neurorehabilitation, to encourage the use of vision for guiding limb movements post-stroke (Pumpa et al., 2015), might be effective when using only one arm or a between-arms asymmetric configuration, but not in the mirror configuration, unless the target is on the body midline (Torre et al., 2013). Since activities of daily living usually involve objects (e.g., grasping), visual feedback on hand position and orientation can often be used to compensate for proprioceptive deficits, as previously suggested (Scalha et al., 2011).

### An Enhanced Patient Stratification

According to the present reasoning, the commonly interpreted proprioceptive deficits might often encompass a larger and in part multi-modal spectrum of dysfunctions. Taking cross-reference processing into account in the assessment may potentially provide a more detailed patient stratification. The deficits may be reclassified into three distinct categories: (P) pure proprioceptive deficits, (C) pure cross-reference processing deficits, and (P+C) mixed proprioceptive and cross-reference processing deficit. Table 2 lists the expected test performance as a function of assessment type and deficit category: although no single test can potentially differentiate these three clinical groups, the different combination of these tests could.

**TABLE 2:** Tasks for which the model predicts an impairment (X) depending on the type of deficit present in patients: (P) deficit of purely proprioceptive origin, (C) cross-reference transformation deficit only, and (C+P) combined deficits.

	**P**	**C**	**P+C**
W-A_*P*_	X		X
W-A_*VP*_			
aB-A_*P*_	X	X	X
aB-A_*VP*_			
sB-A_*P*_	X	X	X
sB-A_*VP*_		X	X
C-M_*P*_	X	X	X
C-M_*VP*_			

This model has limits, since it focuses on stroke deficits in terms of sensory processing. Other factors can interfere with post-stroke performance in the different type of assessments, which are not taken into account by our model, such as age, hand dominance, target memorization, task workspace (Goble, 2010), active or passive reaching (Gurari et al., 2017), position or movement sense (Semrau et al., 2019). However, it provides a framework which reconciles apparently contradictory results from proprioceptive assessments (Torre et al., 2013; Dos Santos et al., 2015; Contu et al., 2017; Gurari et al., 2017; Rinderknecht et al., 2018; Herter et al., 2019; Ingemanson et al., 2019) and from visual compensation tests (Darling et al., 2008; Scalha et al., 2011; Torre et al., 2013; Semrau et al., 2018; Herter et al., 2019), and it adequately predicts tendencies which fit experimental data (Figure 6).

According to the predicted effect of the three type of deficits (P, C, and P+C) on the tests results (Table 2 and Figure 6), the best candidates for stratifying patients, among the assessments that are currently used, would be the combined use of the W-A_*P*_ task (eyes closed) and a sB-A_*VP*_ task (mirror, with visual feedback). Together, these two complementary assessments may help to better stratify patients. In addition to these two methods, adding visual feedback in common proprioceptive tasks (Scalha et al., 2011; Torre et al., 2013; Semrau et al., 2018; Herter et al., 2019; Marini et al., 2019), or using graphesthesia, shape or length discrimination (Van de Winckel et al., 2012; De Diego et al., 2013; Turville et al., 2017) or reaching to visual targets with the unseen hand (Tagliabue and McIntyre, 2011; Elangovan et al., 2019) could help to further explore the complexity of sensorimotor deficits. In the future, to help explore this complexity, robot-assisted tests may enter clinical routine: the tasks are relatively easy and rapid, and 2D robotic manipulators are affordable (Contu et al., 2017; Rinderknecht et al., 2018). Moreover, robotic devices can overcome major limits of current clinical assessment: a quantitative measurement, without ceiling or floor effect, allowing for a more reliable, precise and reproducible evaluation of proprioceptive deficits (Dukelow et al., 2010; Lambercy et al., 2011; Simo et al., 2014; Dos Santos et al., 2015; Contu et al., 2017; Semrau et al., 2017; Deblock-Bellamy et al., 2018; Rinderknecht et al., 2018; Ingemanson et al., 2019).

The proposed stratification of patients may also provide insights about the neural correlates. We would expect that lesions of different brain areas would correspond to the three different categories of deficits. Hypothetically, and informed by the reviewed brain-mapping literature, injury affecting S1 may primarily relate to purely proprioceptive deficits, whereas lesions in the PPC and STG may cause deficits in the ability of performing cross-reference transformations. Patients with mixed deficits would likely tend to have larger lesions affecting both proprioceptive and associative areas. Further lesion-symptom studies examining the correlation of brain lesions in different categories of tasks may offer better identification of brain structures in relation to proprioception or cross-reference processing.

### Application for a More Personalized Rehabilitation Approach

A more accurate stratification of post-stroke patients suffering from proprioceptive deficits should be relevant also for rehabilitation protocols. Given that sensory recovery is a predictor for motor and functional recovery (Bolognini et al., 2016), training of proprioception and cross-reference processing may be key to improve recovery. Currently the effectiveness of sensory rehabilitation is rather weak (Doyle et al., 2010; Findlater and Dukelow, 2017), in part due to heterogeneity in interventions, in outcomes measures (Doyle et al., 2010), and in the precision and reliability of the assessment (Findlater and Dukelow, 2017). A more accurate diagnostic stratification would potentially allow for sensory rehabilitation interventions targeting either proprioception alone, cross-reference processing alone, or both of them; although this would need validation. Adequate training needs to match the symptoms: training restricted to the proprioceptive modality may not address dysfunction in cross-modal processing, and vice versa. Table 3 summarizes hypothetical treatment options based on the present novel stratification of patients with specific deficits. A more accurate assessment of the different sensory functions could also provide a better assessment of the progress made during rehabilitation.

**TABLE 3:** Possible strategies for differential rehabilitation methods depending on the observed sensory deficit: proprioceptive (P), cross-reference (C), and combined (P+C) deficits.

	**P**	**C**	**P+C**
Proprioceptive, within-arm training	X		X
Proprioceptive, between-arm training		X	X
Cross-modal training		X	X
Visual compensation (matching spatial location)	X	X	X
Visual compensation (mirror configuration)	X		

*Xs identify appropriate rehabilitation methods.*

## Conclusion

Proprioception is a prerequisite for normal hand function, in particular for reaching, grasping and object manipulation. Using a theoretical approach, based on statistical models of optimal multi-sensory integration, we have reinterpreted post-stroke proprioceptive deficits, as well as the ability of patients to visually compensate for their deficit. The present analyses highlight that proprioceptive control of the hand may be largely affected by the inability to perform cross-reference transformations, that is to process proprioceptive information in order to encode it, not only in joint space, but also in alternative (often visual) reference frames. This finding allowed us to propose an improved classification of post-stroke deficits, which distinguishes between deficits of the proprioceptive system *per se*, deficits of cross-reference processing, and the combined deficits of the former two. This distinction could lead to a new stratification of stroke patients and may result in more personalized rehabilitation plans.

## Data Availability Statement

The manuscript presents only experimental data from the literature. All datasets generated for this study are included in the article/Supplementary Material, further inquiries can be directed to the corresponding author/s.

## Author Contributions

MT conceptualized the topic of the manuscript. JB-E wrote the initial draft of the manuscript and prepared the figures. All authors contributed to writing the manuscript, and read and approved the submitted version.

## Conflict of Interest

The authors declare that the research was conducted in the absence of any commercial or financial relationships that could be construed as a potential conflict of interest.

## Footnotes

1 Nottingham Sensory Assessment.

2 Fugl-Meyer Assessment for the Upper Extremity.

3 Rivermead Assessment of Somatosensory Performance.

4 We choose here to refer to this group of tasks as “between-arms,” and not bimanual, as the two arms are not used together to sense and move to the target. In contrast, tasks involving only one arm will be referred as “within-arm.”
