# Supplementary material for: Multisensory Integration in Stroke Patients: A Theoretical Approach to Reinterpret Upper-Limb Proprioceptive Deficits and Visual Compensation
Source: Front Neurosci. 2021 Apr 7;15:646698. doi: 10.3389/fnins.2021.646698 (PMC8058201; doi:10.3389/fnins.2021.646698)
Supplement: Supplementary file 1 [file Data_Sheet_1.pdf]

frontiers

Supplementary Material

1 Equations for optimal sensory weighting, case with 2 reference frames

The motor vector Δ_i is defined by the weighted sum of the sensory estimates in the visual (ΔV_i) and proprioceptive (ΔP_i) modality:

$$\Delta_i = w_{\Delta P} \cdot \Delta P_i + w_{\Delta V} \cdot \Delta V_i \qquad \textbf{(S1)}$$

with $w_{\Delta P}$ and $w_{\Delta P}$ the sensory weights for the concurrent target-effector comparisons in the proprioceptive and visual modality respectively, assuming the constraint $C(w_{\Delta P}, w_{\Delta V})$:

$$C(w_{\Delta P}, w_{\Delta V}) = w_{\Delta P} + w_{\Delta V} - 1 = 0 \qquad \textbf{(S2)}$$

Without loss of generality, let Δ have a mean of zero. The variance of Δ is:

$$\sigma_\Delta^2 = \frac{1}{n}\sum_{i=1}^{n}(\Delta_i)^2 \qquad \textbf{(S3)}$$

$$\sigma_\Delta^2 = \frac{1}{n}\sum_{i=1}^{n}(w_{\Delta P} \cdot \Delta P_i + w_{\Delta V} \cdot \Delta V_i)^2 \qquad \textbf{(S4)}$$

$$\sigma_\Delta^2 = (w_{\Delta P})^2 \cdot \frac{1}{n}\sum_{i=1}^{n}(\Delta P_i)^2 + (w_{\Delta V})^2 \cdot \frac{1}{n}\sum_{i=1}^{n}(\Delta V_i)^2 + 2 \cdot w_{\Delta P} \cdot w_{\Delta V} \cdot \frac{1}{n}\sum_{i=1}^{n}\Delta P_i \cdot \Delta V_i \qquad \textbf{(S5)}$$

$$\sigma_\Delta^2(w_{\Delta P}, w_{\Delta V}) = {w_{\Delta P}}^2 \cdot \sigma_{\Delta P}^2 + {w_{\Delta V}}^2 \cdot \sigma_{\Delta V}^2 + 2 \cdot w_{\Delta P} \cdot w_{\Delta V} \cdot cov(\Delta P, \Delta V) \qquad \textbf{(S6)}$$

In order to optimize the sensory weighting, the sensory weights $w_{\Delta P}$ and $w_{\Delta P}$ should be defined to minimize the motor vector's variance σ_Δ^2, under the constraint (S2). We use Lagrange Multiplier technique to minimize the function $\sigma_\Delta^2(w_{\Delta P}, w_{\Delta V})$ with the constraint $C(w_{\Delta P}, w_{\Delta V}) = 0$. We need to solve the following equation system, with λ as the Lagrange multiplier:

$$\begin{cases} \nabla\sigma_\Delta^2(w_{\Delta P}, w_{\Delta V}) = \lambda \cdot \nabla C(w_{\Delta P}, w_{\Delta V}) \\ C(w_{\Delta P}, w_{\Delta V}) = 0 \end{cases} \qquad \textbf{(S7)}$$

$$\begin{cases} \frac{\partial\sigma_\Delta^2(w_{\Delta P}, w_{\Delta V})}{\partial w_{\Delta P}} = \lambda \cdot \frac{\partial C(w_{\Delta P}, w_{\Delta V})}{\partial w_{\Delta P}} \\ \frac{\partial\sigma_\Delta^2(w_{\Delta P}, w_{\Delta V})}{\partial w_{\Delta V}} = \lambda \cdot \frac{\partial C(w_{\Delta P}, w_{\Delta V})}{\partial w_{\Delta V}} \\ w_{\Delta P} + w_{\Delta V} - 1 = 0 \end{cases} \qquad \textbf{(S8)}$$

$$\begin{cases} 2 \cdot w_{\Delta P} \cdot \sigma_{\Delta P}^2 + 2 \cdot w_{\Delta V} \cdot cov(\Delta P, \Delta V) = \lambda \\ 2 \cdot w_{\Delta V} \cdot \sigma_{\Delta V}^2 + 2 \cdot w_{\Delta P} \cdot cov(\Delta P, \Delta V) = \lambda \\ w_{\Delta P} + w_{\Delta V} - 1 = 0 \end{cases} \qquad \textbf{(S9)}$$

which gives the solutions:

$$w_{\Delta P} = \frac{\sigma_{\Delta V}^2 - cov(\Delta P, \Delta V)}{\sigma_{\Delta P}^2 + \sigma_{\Delta V}^2 - 2 \cdot cov(\Delta P, \Delta V)}$$
$$w_{\Delta V} = \frac{\sigma_{\Delta P}^2 - cov(\Delta P, \Delta V)}{\sigma_{\Delta P}^2 + \sigma_{\Delta V}^2 - 2 \cdot cov(\Delta P, \Delta V)} \quad \textbf{(S10)}$$

Replacing $w_{\Delta P}$ and $w_{\Delta V}$ in equation S6 gives the variance of the optimal motor vector σ_{Δ}^2:

$$\sigma_{\Delta}^2 = \frac{\sigma_{\Delta P}^2 \cdot \sigma_{\Delta V}^2 - cov(\Delta P, \Delta V)^2}{\sigma_{\Delta P}^2 + \sigma_{\Delta V}^2 - 2 \cdot cov(\Delta P, \Delta V)} \quad \textbf{(S11)}$$

2 Equations for optimal sensory weighting, case with 4 reference frames

Following the same reasoning, to find the sensory weights $w_{\Delta J}$, $w_{\Delta ExJ}$, $w_{\Delta R}$, and $w_{\Delta ExR}$ for the joint-centered (*J*), extra-joint (*ExJ*), retino-centered (*R*) and extra-retinal (*ExR*) reference-frames respectively, we need to solve the following system of equations:

$$\begin{cases} \nabla\sigma_{\Delta}^2(w_{\Delta J}, w_{\Delta ExJ}, w_{\Delta R}, w_{\Delta ExR}) = \lambda \cdot \nabla C\big(w_{\Delta J}, w_{\Delta ExJ}, w_{\Delta R}, w_{\Delta ExR}\big) \\ C\big(w_{\Delta J}, w_{\Delta ExJ}, w_{\Delta R}, w_{\Delta ExR}\big) = 0 \end{cases} \quad \textbf{(S12)}$$

The general formulation for the variance of the optimal motor vector estimate weights is:

$$\begin{aligned} \sigma_{\Delta}^2 = \; & {w_{\Delta J}}^2 \cdot \sigma_{\Delta J}^2 + {w_{\Delta ExJ}}^2 \cdot \sigma_{\Delta ExJ}^2 + {w_{\Delta R}}^2 \cdot \sigma_{\Delta R}^2 + {w_{\Delta ExR}}^2 \cdot \sigma_{\Delta ExR}^2 \\ & +2 \cdot w_{\Delta J} \cdot w_{\Delta ExJ} \cdot cov(\Delta J, \Delta ExJ) \\ & +2 \cdot w_{\Delta J} \cdot w_{\Delta R} \cdot cov(\Delta J, \Delta R) \\ & +2 \cdot w_{\Delta J} \cdot w_{\Delta ExR} \cdot cov(\Delta J, \Delta ExR) \\ & +2 \cdot w_{\Delta ExJ} \cdot w_{\Delta R} \cdot cov(\Delta ExJ, \Delta R) \\ & +2 \cdot w_{\Delta ExJ} \cdot w_{\Delta ExR} \cdot cov(\Delta ExJ, \Delta ExR) \\ & +2 \cdot w_{\Delta R} \cdot w_{\Delta ExR} \cdot cov(\Delta R, \Delta ExR) \end{aligned} \quad \textbf{(S13)}$$

The equation S12 thus becomes:

$$\begin{cases} 2 \cdot w_{\Delta J} \cdot \sigma_{\Delta J}^2 + 2 \cdot w_{\Delta ExJ} \cdot cov(\Delta J, \Delta ExJ) + 2 \cdot w_{\Delta R} \cdot cov(\Delta J, \Delta R) + 2 \cdot w_{\Delta ExR} \cdot cov(\Delta J, \Delta ExR) = \lambda \\ 2 \cdot w_{\Delta ExJ} \cdot \sigma_{\Delta ExJ}^2 + 2 \cdot w_{\Delta J} \cdot cov(\Delta J, \Delta ExJ) + 2 \cdot w_{\Delta R} \cdot cov(\Delta ExJ, \Delta R) + 2 \cdot w_{\Delta ExR} \cdot cov(\Delta ExJ, \Delta ExR) = \lambda \\ 2 \cdot w_{\Delta R} \cdot \sigma_{\Delta R}^2 + 2 \cdot w_{\Delta J} \cdot cov(\Delta J, \Delta R) + 2 \cdot w_{\Delta ExJ} \cdot cov(\Delta ExJ, \Delta R) + 2 \cdot w_{\Delta ExR} \cdot cov(\Delta R, \Delta ExR) = \lambda \\ 2 \cdot w_{\Delta ExR} \cdot \sigma_{\Delta ExR}^2 + 2 \cdot w_{\Delta J} \cdot cov(\Delta J, \Delta ExR) + 2 \cdot w_{\Delta ExJ} \cdot cov(\Delta ExJ, \Delta ExR) + 2 \cdot w_{\Delta R} \cdot cov(\Delta R, \Delta ExR) = \lambda \\ w_{\Delta J} + w_{\Delta ExJ} + w_{\Delta R} + w_{\Delta ExR} - 1 = 0 \end{cases} \quad \textbf{(S14)}$$

The general solution for the sensory weights $w_{\Delta J}$, $w_{\Delta ExJ}$, $w_{\Delta R}$, and $w_{\Delta ExR}$ is too large to be printed, but is easily calculable using Matlab® (R2019b, with the Symbolic Toolbox).

3 Task specific solutions

Using the general equations for the 4 sensory weights associated to the 4 reference-frames, we can compute the sensory weights for each reference-frame for a given proprioceptive or visuo-proprioceptive task.

3.1 Within-arm proprioceptive tasks (W-A$_P$)

The variance for the 4 concurrent target-effector comparisons are described in the main text (equations 8). The covariances between the concurrent comparisons in the 4 reference frames are:

$$cov(\Delta J, \Delta ExJ) = cov(\Delta J, \Delta R) = cov(\Delta J, \Delta ExR) = cov(\Delta ExJ, \Delta R) = cov(\Delta ExJ, \Delta ExR) = \sigma_J^2 + \sigma_J^2$$
$$cov(\Delta R, \Delta ExR) = \sigma_J^2 + \sigma_{J\to R}^2 + \sigma_J^2 + \sigma_{J\to R}^2 \quad \textbf{(S15)}$$

Replacing these terms (equations 8, and equations S15) in the system S14 gives the optimal weights:

$$\begin{aligned} w_{\Delta J} &= 1 \\ w_{\Delta ExJ} &= 0 \\ w_{\Delta R} &= 0 \\ w_{\Delta ExR} &= 0 \end{aligned} \quad \textbf{(S16)}$$

Using this optimal set of weights (equations S16), the variances (equations 8) and covariances (equations S15), we obtain the variance for the optimal motor vector by replacing these terms in equation S13 as described in the manuscript (equation 9).

3.2 Asymmetric between-arms proprioceptive tasks (aB-A$_P$)

The variance for the 4 concurrent target-effector comparisons are described in the main text (equations 10). The covariances between the concurrent comparisons in the 4 reference frames are:

$$cov(\Delta J, \Delta ExJ) = cov(\Delta J, \Delta R) = cov(\Delta J, \Delta ExR) = cov(\Delta ExJ, \Delta R) = cov(\Delta ExJ, \Delta ExR) = \sigma_{J_r}^2 + \sigma_{J_l}^2$$
$$cov(\Delta R, \Delta ExR) = \sigma_{J_r}^2 + \sigma_{J_r\to R}^2 + \sigma_{J_l}^2 + \sigma_{J_l\to R}^2 \quad \textbf{(S17)}$$

For the asymmetric configuration, since the joint signals are not directly comparable, we consider $\sigma_{J_{r\leftrightarrow l}}^2 \to \infty$. Moreover, because the hand and the target have the same position in space, the visual reconstruction of hand and target are directly comparable. Therefore we consider $\sigma_{M_{r\leftrightarrow l}}^2 \to 0$. Replacing these terms (equations 10, and equations S17) in the system S14 gives the optimal weights:

$$\begin{aligned} w_{\Delta J} &\to 0 \\ w_{\Delta ExJ} &\to \frac{\sigma_{J\to R}^2}{\sigma_{J\to R}^2 + \sigma_{J\to ExJ}^2} \\ w_{\Delta R} &\to \frac{\sigma_{J\to ExJ}^2}{\sigma_{J\to R}^2 + \sigma_{J\to ExJ}^2} \\ w_{\Delta ExR} &\to 0 \end{aligned} \quad \textbf{(S18)}$$

Using this optimal set of weights (equations S18), the variances (equations 10) and covariances (equations S17), we obtain the variance for the optimal motor vector by replacing these terms in equation S13 as described in the manuscript (equation 11).

3.3 Symmetric between-arms proprioceptive tasks (sB-A$_P$)

The variance for the 4 concurrent target-effector comparisons are described in the main text (equations 12). The covariances between the concurrent comparisons in the 4 reference frames are:

$$cov(\Delta J, \Delta ExJ) = cov(\Delta J, \Delta R) = cov(\Delta J, \Delta ExR) = cov(\Delta ExJ, \Delta R) = cov(\Delta ExJ, \Delta ExR) = \sigma_{J_r}^2 + \sigma_{J_l}^2$$
$$cov(\Delta R, \Delta ExR) = \sigma_{J_r}^2 + \sigma_{J_r \to R}^2 + \sigma_{J_l}^2 + \sigma_{J_l \to R}^2 \quad \textbf{(S19)}$$

For the symmetric configuration, since the analogous joint signals from both arms are theoretically comparable, we consider $\sigma_{J_{r \leftrightarrow l}}^2 \to 0$. Moreover, because the hand and the target do not have the same position in space, the visual reconstruction of hand and target on the retina are not directly comparable. Therefore we consider $\sigma_{R,Mir}^2 \to \infty$. Replacing these terms (equations 12, and equations S19) in the system S14 gives the optimal weights:

$$\begin{aligned} w_{\Delta J} &\to 1 \\ w_{\Delta ExJ} &\to 0 \\ w_{\Delta R} &\to 0 \\ w_{\Delta ExR} &\to 0 \end{aligned} \quad \textbf{(S20)}$$

However, considering a patient with brain lesions affecting the ability to perform easily inter-hemispheric transformations, we cannot postulate that $\sigma_{J_{r \leftrightarrow l}}^2 \to 0$. The set of weights becomes:

$$\begin{aligned} w_{\Delta J} &\to \frac{\sigma_{J \to ExJ}^2(\sigma_{J \to R}^2 + \sigma_{R \to ExR}^2)}{(\sigma_{J \to ExJ}^2 + \sigma_{J r \leftrightarrow l}^2)(\sigma_{J \to R}^2 + \sigma_{R \to ExR}^2) + \sigma_{J r \leftrightarrow l}^2 \sigma_{J \to ExJ}^2} \\ w_{\Delta ExJ} &\to \frac{\sigma_{J r \leftrightarrow l}^2(\sigma_{J \to R}^2 + \sigma_{R \to ExR}^2)}{(\sigma_{J \to ExJ}^2 + \sigma_{J r \leftrightarrow l}^2)(\sigma_{J \to R}^2 + \sigma_{R \to ExR}^2) + \sigma_{J r \leftrightarrow l}^2 \sigma_{J \to ExJ}^2} \\ w_{\Delta R} &\to 0 \\ w_{\Delta ExR} &\to \frac{\sigma_{J \to ExJ}^2 \sigma_{J r \leftrightarrow l}^2}{(\sigma_{J \to ExJ}^2 + \sigma_{J r \leftrightarrow l}^2)(\sigma_{J \to R}^2 + \sigma_{R \to ExR}^2) + \sigma_{J r \leftrightarrow l}^2 \sigma_{J \to ExJ}^2} \end{aligned} \quad \textbf{(S21)}$$

Using this optimal sets of weights (equations S20 and S21), the variances (equations 12) and covariances (equations S19), we obtain the variance for the optimal motor vector by replacing these terms in equation S13 as described in the manuscript (equation 13).

3.4 Cross-modal task (C-M$_P$)

The variance for the 4 concurrent target-effector comparisons are described in the main text (equations 14). The covariances between the concurrent comparisons in the 4 reference frames are:

$$\begin{aligned} &cov(\Delta J, \Delta ExJ) = \sigma_R^2 + \sigma_{R \to J}^2 + \sigma_J^2 \\ &cov(\Delta R, \Delta ExR) = \sigma_R^2 + \sigma_J^2 + \sigma_{J \to R}^2 \\ &cov(\Delta J, \Delta R) = cov(\Delta J, \Delta ExR) = cov(\Delta ExJ, \Delta R) = cov(\Delta ExJ, \Delta ExR) = \sigma_R^2 + \sigma_J^2 \end{aligned} \quad \textbf{(S22)}$$

Replacing these terms (equations 14, and equations S22) in the system S14 gives the optimal weights:

$$\begin{aligned} w_{\Delta J} &= \frac{\sigma_{J \to R}^2}{\sigma_{J \to R}^2 + \sigma_{R \to J}^2} \\ w_{\Delta ExJ} &= 0 \\ w_{\Delta R} &= \frac{\sigma_{R \to J}^2}{\sigma_{J \to R}^2 + \sigma_{R \to J}^2} \\ w_{\Delta ExR} &= 0 \end{aligned} \quad \textbf{(S23)}$$

Using this optimal set of weights (equations S23), the variances (equations 14) and covariances (equations S22), and considering $\sigma^2_{R\to J} = \sigma^2_{J\to R} = \sigma^2_{J\leftrightarrow R}$, we obtain the variance for the optimal motor vector by replacing these terms in equation S13 as described in the main text (equation 15).

3.5 Within-arm visuo-proprioceptive tasks (W-A$_{VP}$)

The variance for the 4 concurrent target-effector comparisons are described in the main text (equations 16). The covariances between the concurrent comparisons in the 4 reference frames are:

$$\begin{aligned} &cov(\Delta J, \Delta ExJ) = \sigma_J^2 + \sigma_J^2 \\ &cov(\Delta R, \Delta ExR) = \sigma_R^2 + \sigma_R^2 \\ &cov(\Delta J, \Delta R) = cov(\Delta J, \Delta ExR) = cov(\Delta ExJ, \Delta R) = cov(\Delta ExJ, \Delta ExR) = 0 \end{aligned} \quad \textbf{(S24)}$$

Replacing these terms (equations 16, and equations S24) in the system S14 gives the optimal weights:

$$\begin{aligned} w_{\Delta J} &= \frac{\sigma_R^2}{\sigma_J^2+\sigma_R^2} \\ w_{\Delta ExJ} &= 0 \\ w_{\Delta R} &= \frac{\sigma_J^2}{\sigma_J^2+\sigma_R^2} \\ w_{\Delta ExR} &= 0 \end{aligned} \quad \textbf{(S25)}$$

Using this optimal set of weights (equations S25), the variances (equations 16) and covariances (equations S24), we obtain the variance for the optimal motor vector by replacing these terms in equation S13 as described in the manuscript (equation 17).

3.6 Asymmetric between-arms visuo-proprioceptive task (aB-A$_{VP}$)

The variance for the 4 concurrent target-effector comparisons are described in the main text (equations 18). The covariances between the concurrent comparisons in the 4 reference frames are:

$$\begin{aligned} &cov(\Delta J, \Delta ExJ) = \sigma_{J_r}^2 + \sigma_{J_l}^2 \\ &cov(\Delta R, \Delta ExR) = \sigma_R^2 + \sigma_R^2 \\ &cov(\Delta J, \Delta R) = cov(\Delta J, \Delta ExR) = cov(\Delta ExJ, \Delta R) = cov(\Delta ExJ, \Delta ExR) = 0 \end{aligned} \quad \textbf{(S26)}$$

For the asymmetric configuration, since the joint signals are not directly comparable, we consider $\sigma^2_{J_{r\leftrightarrow l}} \to \infty$. However, because the hand and the target have the same position in space, a direct visual comparison is possible. We consider $\sigma^2_{M_{r\leftrightarrow l}} = 0$. Replacing these terms (equations 18, and equations S26) in the system S14 gives the optimal weights:

$$\begin{aligned} w_{\Delta J} &\to 0 \\ w_{\Delta ExJ} &\to \frac{2\sigma_R^2}{2\sigma_R^2 + \sigma_{J_r}^2 + \sigma_{J_l}^2 + 2\sigma_{J\to ExJ}^2} \\ w_{\Delta R} &\to \frac{\sigma_{J_r}^2 + \sigma_{J_l}^2 + 2\sigma_{J\to ExJ}^2}{2\sigma_R^2 + \sigma_{J_r}^2 + \sigma_{J_l}^2 + 2\sigma_{J\to ExJ}^2} \\ w_{\Delta ExR} &\to 0 \end{aligned} \quad \textbf{(S27)}$$

Using this optimal set of weights (equations S27), the variances (equations 18) and covariances (equations S26), we obtain the variance for the optimal motor vector by replacing these terms in equation S13 as described in the main text (equation 19).

3.7 Symmetric between-arms visuo-proprioceptive task (sB-A$_{VP}$)

The variance for the 4 concurrent target-effector comparisons are described in the main text (equations 20). The covariances between the concurrent comparisons in the 4 reference frames are:

$$\begin{aligned}
&cov(\Delta J, \Delta ExJ) = \sigma_{J_r}^2 + \sigma_{J_l}^2 \\
&cov(\Delta R, \Delta ExR) = \sigma_R^2 + \sigma_R^2 \\
&cov(\Delta J, \Delta R) = cov(\Delta J, \Delta ExR) = cov(\Delta ExJ, \Delta R) = cov(\Delta ExJ, \Delta ExR) = 0
\end{aligned} \tag{S28}$$

For the symmetric configuration, since the analogous joint signals from both arms are theoretically comparable, we consider $\sigma_{J_{r\leftrightarrow l}}^2 \to 0$. Moreover, because the hand and the target do not have the same position in space, the visual reconstruction of hand and target on the retina are not directly comparable. Therefore we consider $\sigma_{R,Mir}^2 \to \infty$. Replacing these terms (equations 20, and equations S28) in the system S14 gives the optimal weights:

$$\begin{aligned}
w_{\Delta J} &\to \frac{2\cdot(\sigma_R^2+\sigma_{R\leftrightarrow ExR}^2)}{\sigma_{J_r}^2+\sigma_{J_l}^2+2\cdot\sigma_R^2+2\cdot\sigma_{R\leftrightarrow ExR}^2} \\
w_{\Delta ExJ} &\to 0 \\
w_{\Delta R} &\to 0 \\
w_{\Delta ExR} &\to \frac{\sigma_{J_r}^2+\sigma_{J_l}^2}{\sigma_{J_r}^2+\sigma_{J_l}^2+2\cdot\sigma_R^2+2\cdot\sigma_{R\leftrightarrow ExR}^2}
\end{aligned} \tag{S29}$$

However, considering a patient with brain lesions affecting the ability to perform easily cross-reference transformations, we cannot postulate that $\sigma_{J_r\leftrightarrow J_l}^2 \to 0$. The set of weights becomes:

$$\begin{aligned}
w_{\Delta J} &\to \frac{2\cdot\sigma_{J\to ExJ}^2(\sigma_R^2+\sigma_{R\to ExR}^2)}{(\sigma_{J\to ExJ}^2+\sigma_{J_{r\leftrightarrow l}}^2)\cdot(\sigma_{J_r}^2+\sigma_{J_l}^2+2\cdot\sigma_R^2+2\cdot\sigma_{R\to ExR}^2)+2\cdot\sigma_{J\to ExJ}^2\cdot\sigma_{J_{r\leftrightarrow l}}^2} \\
w_{\Delta ExJ} &\to \frac{2\cdot\sigma_{J_{r\leftrightarrow l}}^2(\sigma_R^2+\sigma_{R\to ExR}^2)}{(\sigma_{J\to ExJ}^2+\sigma_{J_{r\leftrightarrow l}}^2)\cdot(\sigma_{J_r}^2+\sigma_{J_l}^2+2\cdot\sigma_R^2+2\cdot\sigma_{R\to ExR}^2)+2\cdot\sigma_{J\to ExJ}^2\cdot\sigma_{J_{r\leftrightarrow l}}^2} \\
w_{\Delta R} &\to 0 \\
w_{\Delta ExR} &\to \frac{(\sigma_{J_r}^2+\sigma_{J_l}^2)\cdot(\sigma_{J\to ExJ}^2+\sigma_{J_{r\leftrightarrow l}}^2)+2\cdot\sigma_{J\to ExJ}^2\cdot\sigma_{J_{r\leftrightarrow l}}^2}{(\sigma_{J\to ExJ}^2+\sigma_{J_{r\leftrightarrow l}}^2)\cdot(\sigma_{J_r}^2+\sigma_{J_l}^2+2\cdot\sigma_R^2+2\cdot\sigma_{R\to ExR}^2)+2\cdot\sigma_{J\to ExJ}^2\cdot\sigma_{J_{r\leftrightarrow l}}^2}
\end{aligned} \tag{S30}$$

Using this optimal set of weights (equations S29 and S30), the variances (equations 20) and covariances (equations S28), we obtain the variance for the optimal motor vector by replacing these terms in equation S13 as described in the main text (equation 21).

3.8 Cross-modal task, with full visual feedback (C-M$_{VP}$)

The variance for the 4 concurrent target-effector comparisons are described in the main text (equations 22). The covariances between the concurrent comparisons in the 4 reference frames are:

$$cov(\Delta J, \Delta ExJ) = \sigma_R^2 + \sigma_{R\rightarrow J}^2 + \sigma_J^2$$
$$cov(\Delta R, \Delta ExR) = \sigma_R^2 + \sigma_R^2 \quad \textbf{(S31)}$$
$$cov(\Delta J, \Delta R) = cov(\Delta J, \Delta ExR) = cov(\Delta ExJ, \Delta R) = cov(\Delta ExJ, \Delta ExR) = \sigma_R^2$$

Replacing these terms (equations 22, and equations S31) in the system S14 gives the optimal weights:

$$\begin{aligned} w_{\Delta J} &= \frac{\sigma_R^2}{\sigma_J^2+\sigma_{R\rightarrow J}^2+\sigma_R^2} \\ w_{\Delta ExJ} &= 0 \\ w_{\Delta R} &= \frac{\sigma_J^2+\sigma_{R\rightarrow J}^2}{\sigma_J^2+\sigma_{R\rightarrow J}^2+\sigma_R^2} \\ w_{\Delta ExR} &= 0 \end{aligned} \quad \textbf{(S32)}$$

Using this optimal set of weights (equations S32), the variances (equations 22) and covariances (equations S31), we obtain the variance for the optimal motor vector by replacing these terms in equation S13 as described in the main text (equation 23).

4 Visual compensation

To illustrate the ability of patients to compensate for their proprioceptive deficits with vision, we can express, for each task category, the ratio between the variance of the motor vector in the visuo-proprioceptive task and the proprioceptive task. We analyzed the contribution of the sensory deficit nature (either pure proprioceptive deficit (P), pure cross-reference processing deficit (C) or mixed deficit (P+C)) to the relative performance of task with and without vision.

4.1 Within-arm tasks

For the W-A task (see manuscript, equations 9 and 17), the performance of the proprioceptive task, relative to the visuo-proprioceptive task, is only affected by the proprioceptive noisiness (represented by the additional joint signal noise N_J): the more proprioception is affected, the less the proprioceptive task (W-A_P) is precise relative to the visuo-proprioceptive task (W-A_{VP}). This means patients can always improve performance with visual feedback to compensate for the proprioceptive deficit in the W-A_P task. Moreover, the stronger the proprioceptive deficit, the larger will be the advantage provided by using visual information (Supplementary Figure 1).

4.2 Asymmetric between-arms tasks

Similarly, for the aB-A task (see manuscript, equations 11 and 19), the performance of the proprioceptive task, relative to the visuo-proprioceptive task, is affected by both the proprioceptive cross-reference transformations noisiness (represented by the deficit factors N_J and N_T respectively): the more proprioception or cross-reference transformations are affected, the less the proprioceptive task (aB-A_P) is precise relative to the visuo-proprioceptive task (aB-A_{VP}). This means patients can always improve performance with visual feedback to compensate for the proprioceptive and cross-reference deficits in the aB-A_P task. As well, the stronger the sensory deficit (proprioceptive, cross-reference or mixed), the larger will be the advantage provided by using visual information (Supplementary Figure 2).

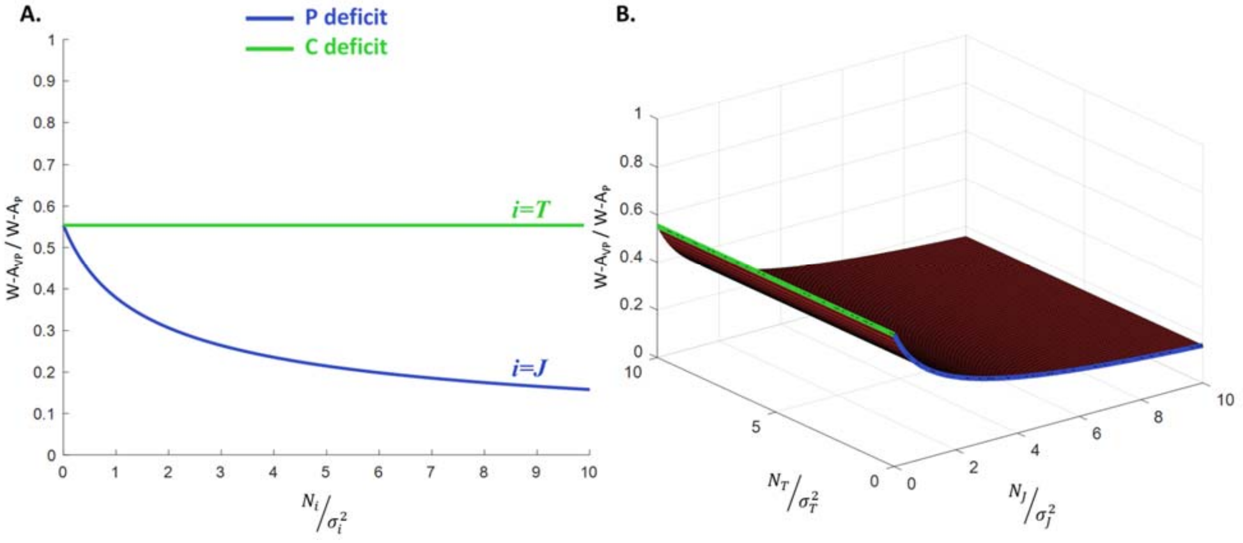

Supplementary Figure 1. Ratio between the variance of the within-arm proprioceptive and visuo-proprioceptive tasks (W-A$_{VP}$/W-A$_P$) as function of the additional noise associated to stroke deficits. **(A)** represents patients with either pure proprioceptive deficit (P: N_J>0 and N_T=0) or pure cross-reference deficits (C: N_J=0 and N_T>0). **(B)** represents patients with mixed (P+C: N_J>0 and N_T>0) deficit. For these plots, we used the values associated to healthy subjects (σ_J^2, σ_R^2 and σ_T^2) obtained with our fitting algorithm (figures 6A and 6B).

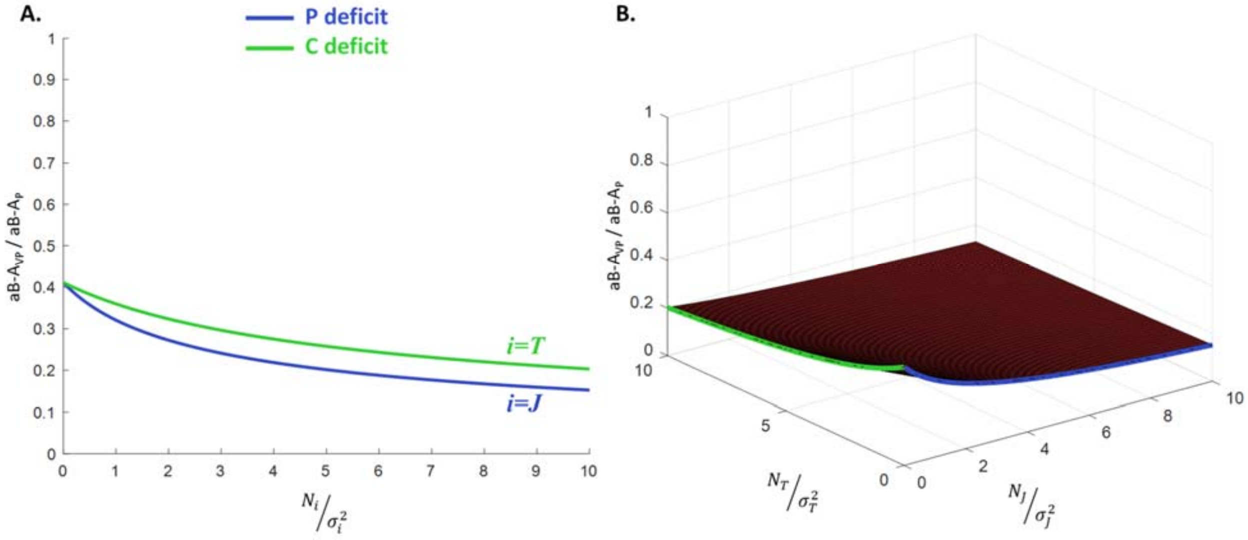

Supplementary Figure 2. Ratio between the variance of the asymmetric between-arms proprioceptive and visuo-proprioceptive tasks (aB-A$_{VP}$/aB-A$_P$) as function of the additional noise associated to stroke deficits. **(A)** represents patients with either pure proprioceptive deficit (P: N_J>0 and N_T=0) or pure cross-reference deficits (C: N_J=0 and N_T>0). **(B)** represents patients with mixed (P+C: N_J>0 and N_T>0) deficit. For these plots, we used the values associated to healthy subjects (σ_J^2, σ_R^2 and σ_T^2) obtained with our fitting algorithm (figures 6A and 6B).

4.3 Symmetric between-arms tasks

For the sB-A task (see manuscript, equations 13 and 21), the performance of the proprioceptive task (sB-AP), relative to the visuo-proprioceptive task (sB-AVP), is also affected by both the proprioceptive and cross-reference transformations noisiness (represented by the deficit factors N_J and N_T respectively). If we consider a patient with pure proprioceptive deficit ($N_J > 0$ and $N_T = 0$), the stronger the proprioceptive deficit, the larger will be the advantage provided by using visual information (Supplementary Figure 3). But, in contrast with the aB-AP tasks, vision hardly compensate for the cross-reference deficit in patients with sensory transformation deficits: the more cross-reference transformations are affected, the less patients can improve with visual feedback. Indeed, the performance in the sB-AVP task tends to the performance in the sB-AP task.

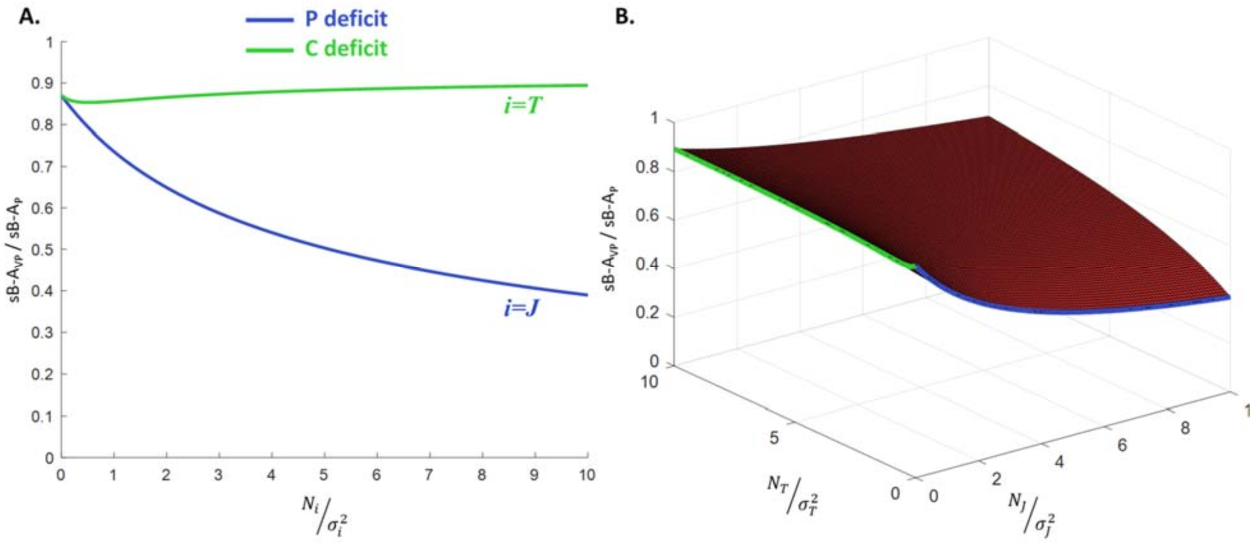

Supplementary Figure 3. Ratio between the variance of the symmetric between-arms proprioceptive and visuo-proprioceptive tasks (sB-AVP/sB-AP) as function of the additional noise associated to stroke deficits. **(A)** represents patients with either pure proprioceptive deficit (P: N_J>0 and N_T=0) or pure cross-reference deficits (C: N_J=0 and N_T>0). **(B)** represents patients with mixed (P+C: N_J>0 and N_T>0) deficit. For these plots, we used the values associated to healthy subjects (σ_J^2, σ_R^2 and σ_T^2) obtained with our fitting algorithm (figures 6A and 6B).

4.4 Cross-modal tasks

For the C-M task (see manuscript, equations 15 and 23), as for the W-A and aB-A tasks, patients can always improve performance with visual feedback to compensate for the proprioceptive or cross-reference deficits. The stronger the sensory deficit (proprioceptive, cross-reference or mixed), the larger will be the advantage provided by using visual information (Supplementary Figure 4).

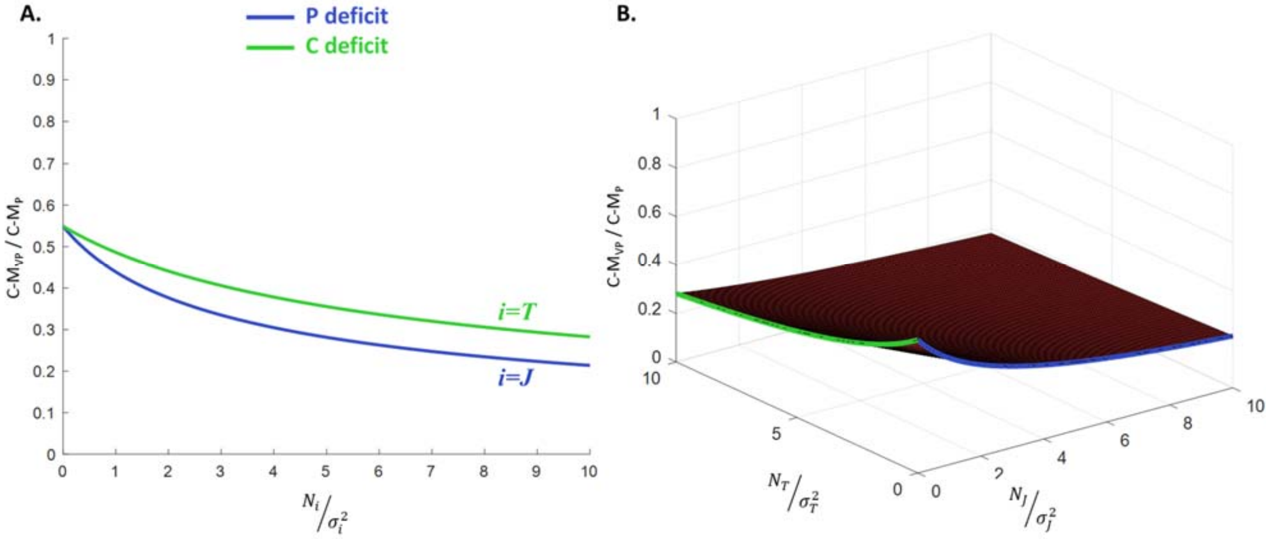

Supplementary Figure 4. Ratio between the variance of the cross-modal proprioceptive and visuo-proprioceptive tasks ($C\text{-}M_{VP}/C\text{-}M_P$) as function of the additional noise associated to stroke deficits. **(A)** represents patients with either pure proprioceptive deficit (P: $N_J>0$ and $N_T=0$) or pure cross-reference deficits (C: $N_J=0$ and $N_T>0$). **(B)** represents patients with mixed (P+C: $N_J>0$ and $N_T>0$) deficit. For these plots, we used the values associated to healthy subjects (σ_J^2, σ_R^2 and σ_T^2) obtained with our fitting algorithm (figures 6A and 6B).

5 Model fitting

5.1 Experimental data used for the fitting

We used 17 data points, extracted from the literature, for fitting our model parameters. The data are shown in Supplementary Table 1 for healthy subjects, and in Supplementary Tables 2, 3 and 4 for patients with P, C and P+C deficits respectively.

The data were most often extracted from graphs. Therefore, its lecture was not always precise. For this reason, we rounded the data at the first digit. The following tables combine absolute and variable errors, as not all selected studies use the same parameter to describe the performance variability.

5.2 Algorithm

For fitting our model to the experimental data, we used Matlab® built-in "fmincon" function (R2019b, with the Optimization Toolbox) to minimize the *l2-norm* of the fitting errors, represented by the following cost function cf:

$$cf = \sum_{i=1}^{n} \left(\sigma_{exp_i} - \sigma_{th_i}\right)^2$$

Where n is the number of data points ($n = 17$), σ_{exp_i} is the normalized variability of the responses for a given task, and σ_{th_i} is the normalized variability for the same task predicted by the model.

In order to avoid data overfitting, the number of independent variables in the model was reduced to six ($v = 6$): the noise of the joint (σ_J^2) and retinal (σ_R^2) signals and the noise associated to sensory transformations (σ_T^2) in healthy subjects; for patients, three terms representing the noise added to the joint signal of the more affected (N_{J_m}) and less affected arm (N_{J_l}) and to the sensory transformations (N_T) due to the stroke lesions.

The number of degrees of freedom, d, of the fitting procedure, which is defined as the difference between the number of data points to be fitted, n, and the number of parameters of the model, v, is therefore:

$$d = n - v = 17 - 6 = 11$$

Supplementary Table 1. Performance variability reported in studies involving healthy subjects. These data points are represented by black squares on the Figure 6 in the main text. The data are normalized with respect to the W-A_P task precision. When the W-A_P was not part of the study (Herter et al. 2019; Cameron and López-Moliner 2015; Khanafer and Cressman 2014; Monaco et al. 2010), a different experimental task, represented by "X", was used as a reference. Then, this ratio was normalized to the W-A_P task. For example, in Herter et al. 2019, the performance in the sB-A_{VP} was first normalized by the performance in the sB-A_P task. Finally, the ratio sB-A_{VP}/sB-A_P was multiplied by the normalized value for the sB-A_P task (sB-A_P/W-A_P). So that sB-A_{VP}/sB-A_P * sB-A_P/W-A_P = sB-A_{VP}/W-A_P corresponds to the variability of the sB-A_{VP} task, normalized by the W-A_P task.

Study \ Task	W-A_P	aB-A_P	sB-A_P	C-M_P	W-A_{VP}	aB-A_{VP}	sB-A_{VP}	C-M_{VP}
Van Beers et al. 1996		X		1.6				
Ernst and Banks 2002	1				0.2			
Butler et al. 2004	1	1.1		1.2				
Monaco et al. 2010		1.9		X		0.4		0.4
Tagliabue and McIntyre 2011	1			1.1				0.6
Torre et al. 2013	1				1			
Khanafer and Cressman 2014		X		1.5				
Cameron and López-Moliner 2015				X				0.8
Arnoux et al. 2017	1	2.3	1.1					
Herter et al. 2019			X				0.9	
Marini et al. 2019	1				0.4			
mean (±SD)	1	1.8±0.6	1.1	1.3±0.2	0.5±0.4	0.4	0.9	0.6±0.2

Supplementary Table 2. Performance variability reported in studies involving patients with proprioceptive only (P) deficits. These data points are represented by blue diamonds on the Figure 6 of the main text. The data are first expressed as a ratio of the performance of healthy subjects in the same study, and then normalized to the W-A_P task precision of healthy participants. The letter "Q" represents qualitative results that were not used for the fitting, but that appear on the Figure 6, as gray rectangles.

Study \ Task	W-A_P	aB-A_P	sB-A_P	C-M_P	W-A_{VP}	aB-A_{VP}	sB-A_{VP}	C-M_{VP}
Scalha et al. 2011						Q		Q
Torre et al. 2013	1.4				0.9			
Dos Santos et al. 2015	2.5							
Contu et al. 2017	1.4							
Rinderknecht et al. 2018	1.9							
Herter et al. 2019			1.8				1.1	
mean (±SD)	1.8±0.5		1.8		0.9		1.1	

Supplementary Table 3. Performance variability reported in the selected studies involving patients with cross-reference only (C) deficits. These data points are represented by green diamonds on the Figure 6. The data are first expressed as a ratio of the performance of healthy subjects in the same study, and then normalized to the W-A_P task precision of healthy participants. The letter "Q" represents qualitative results that were not used for the fitting, but that appear on the Figure 6, as the gray rectangles.

Study \ Task	W-A_P	aB-A_P	sB-A_P	C-M_P	W-A_{VP}	aB-A_{VP}	sB-A_{VP}	C-M_{VP}
Scalha et al. 2011						Q		Q
Gurari et al. 2017	1.2		Q					
mean (±SD)	1.2							

Supplementary Table 4. Performance variability reported in the selected studies involving patients with proprioceptive and cross-reference (P+C) deficits. These data points are represented by red diamonds on the Figure 6. Data points for the sB-A_P and sB-A_{VP} tasks were associated P+C patients, because in the absence of cues allowing a more specific discrimination between C and P+C types of deficits, it is statically more likely that the majority of patients tested in Herter et al. (2019) and Ingemanson et al. (2019) were P+C patients. It cannot be totally excluded, however, that these data could confound C and P+C patients. The data are first expressed as a ratio of the performance of healthy subjects in the same study, and then normalized to the W-A_P task precision of healthy participants. The letter "Q" represents qualitative results that were not used for the fitting, but that appear on the Figure 6, as the gray rectangles.

Study \ Task	W-A_P	aB-A_P	sB-A_P	C-M_P	W-A_{VP}	aB-A_{VP}	sB-A_{VP}	C-M_{VP}
Scalha et al. 2011						Q		Q
Torre et al. 2013	1.4				0.9			
Dos Santos et al. 2015	2.5							
Contu et al. 2017	1.4							
Rinderknecht et al. 2018	1.9							
Herter et al. 2019 (a)			3.0				3.0	
Herter et al. 2019 (b)			3.0				2.1	
Ingemanson et al. 2019			2.5					
mean (±SD)	1.8±0.5		2.8±0.3		0.9		2.5±0.6	

(a) patients who showed no improvement in the sB-A_{VP} task, with respect to the sB-A_P task.
(b) patients who showed only partial improvement in the sB-A_{VP} task, with respect to the sB-A_P task.

5.3 Fitting results

The best fitting between the model predictions and the experimental data is obtained when the six parameters of the model have the following values:

$$\begin{cases} \sigma_J^2 = 0.61 \\ \sigma_R^2 = 0.27 \\ \sigma_T^2 = 1.63 \\ N_{J_m} = 1.08 \\ N_{J_l} = 0.55 \\ N_T = 6.25 \end{cases}$$

The residuals from the fitting procedure are displayed in Supplementary Figure 5. For testing if the residuals are normally distributed, we used the Shapiro-Wilk parametric hypothesis test of composite normality. The statistical test did not reject the normality assumption (W=0.96, p=0.71).

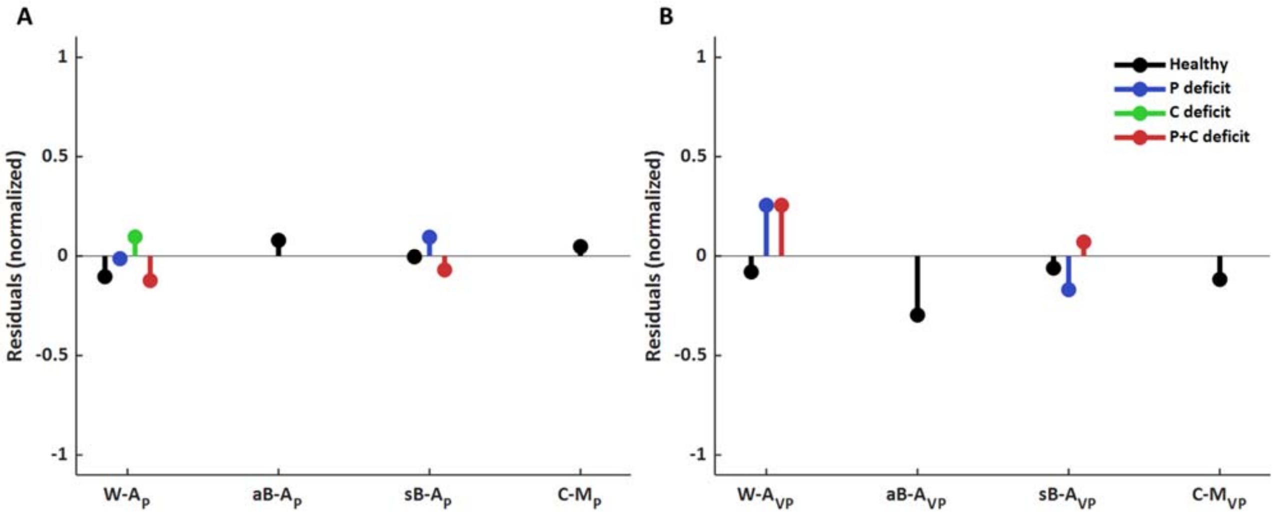

Supplementary Figure 5. Residuals from the fitting procedure, for each sensory condition.

The Root Mean Square (RMS) of the residuals is 0.14.

The adjusted R-Squared (with 11 degrees of freedom) is 0.93, meaning our model accounts for 93% of the total variability in the experimental data.
